# CFD Simulation of Particle-Laden Flow in a 3D Differentially Heated Cavity Using Coarse Large Eddy Simulation

**DOI:** 10.1007/s10494-022-00356-4

**Published:** 2022-08-16

**Authors:** M. A. Sayed, A. Dehbi, M. Hadžiabić, B. Ničeno, K. Mikityuk

**Affiliations:** 1 Paul Scherrer Institut (PSI), 5232 Villigen, Switzerland; 2 Swiss Federal Institute of Technology Lausanne (EPFL), 1015 Lausanne, Switzerland; 3 International University of Sarajevo, 71000 Sarajevo, Bosnia and Herzegovina

**Keywords:** CFD, Turbulence modeling, Heat transfer, Buoyancy-driven flows, Natural convection coarse LES (CLES), Differentially heated cavity (DHC), Particle removal efficiency, Boussinesq approximation, Lagrangian particle tracking

## Abstract

Particulate flow in closed space is involved in many engineering applications. In this paper, the prediction of particle removal is investigated in a thermally driven 3D cavity at turbulent Rayleigh number Ra = 10^9^ using Coarse Large Eddy Simulation (CLES). The depletion dynamics of SiO_2_ aerosol with aerodynamic diameters between 1.4 and 14 µm is reported in an Euler/Lagrange framework. The main focus of this work is therefore to assess the effect of the subgrid-scale motions on the prediction of the particulate flow in a buoyancy driven 3D cavity flow when the mesh resolution is coarse and below optimal LES standards. The research is motivated by the feasibility of modeling more complex particulate flows with reduced CPU cost.

The cubical cavity of 0.7 m side-length is set to have a temperature difference of 39 K between the two facing cold and hot vertical walls. As a first step, the carrier fluid flow was validated by comparing the first and second-moment statistics against both previous well-resolved LES and experimental databases [Kalilainen (J. Aero Sci. 100:73–87, 2016); Dehbi (J. Aero. Sci. 103:67–82, 2017)]. First moment Eulerian statistics show a very good match with the reference data both qualitatively and quantitatively, whereas higher moments show underprediction due to the lesser spatial resolution. In a second step, six particle swarms spanning a wide range of particle Stokes numbers were computed to predict particle depletion. In particular, predictions of 1.4 and 3.5 µm particles were compared to LES and available experimental data. Particles of low inertia i.e. dp < 3.5 µm are more affected by the SGS effects, while bigger ones i.e. dp = 3.5–14 µm exhibit much less grid-dependency. Lagrangian statistics reported in both qualitative and quantitative fashions show globally a very good agreement with reference LES and experimental databases at a fraction of the CPU power needed for optimal LES.

## Introduction

Particle transport in closed spaces is crucial for many engineering, medical and environmental applications, from pathogen flow inside hospital rooms and the transport of air-borne pollutants in clean rooms to radioactive particulate flows in nuclear plants and aircraft-related applications (Morrison et al. 2006; Kam et al. 1998; Wana et al. 2007; Poussou et al. 2010; Jones and Kissane 2000). Having a reliable design-based tool for predicting such flows can significantly improve the process of prototype-based analyses. A typical example is thermally-driven flow in a parallelepiped cavity. Such configuration is referred to as a differentially heated cavity (DHC) where two opposite vertical walls are kept at two different temperatures and the rest of the walls are adiabatically insulated. Over the last few decades, numerous studies have investigated the DHC flow both numerically and experimentally (Vahl Davis 1968; Mallinson and Wahl Davis 1977; Briggs and Jones 1985; Trias et al. 2010a, 2010b, 2007a; Colomer et al. 2004; Baïri et al. 2007; Nansteel and Grief 1984; Frederick and Valencia 1989; Ampofo and Karayiannis 2003; Tian and Karayiannis 2000a, 2000b; Betts and Bokhari 2000; Xin and Quéré 1995; Bejan 1995; Mergui and Penot 1996; Salat et al. 2004). In particular, the first numerical simulation of flow inside a DHC date back to 1968, when De Vahl Davis (1968) conducted a study on 2D square cavities with laminar flow. With the growth of computer power, researchers endeavor 2D and 3D simulations/computations of turbulent flows inside DHC with moderate to high Rayleigh numbers with both large eddy simulation (LES) and direct numerical simulation (DNS), (Kim et al. 2018; Trias et al. 2007b; Puragliesi 2010; Bosshard et al. 2013; Sebilleau et al. 2018; Sayed et al. 2021) among others. Currently, the DHC serves as one of the main benchmarks to validate turbulent buoyancy-driven flows. In this paper, we first focus on the flow prediction before investigating the discrete phase dispersion in a second step.

To properly predict particle dispersion, the underlying carrier flow must be well-predicted. Despite being a simple geometry, DHC poses a challenge for CFD models to accurately capture the flow behavior in a 3D turbulent regime. The main challenge for the Unsteady RANS (URANS) is the simultaneous existence of both laminar and turbulent regions and the underlying transition from laminar to turbulent flow. It is known that eddy-viscosity-based turbulence models cannot capture laminar-turbulence transition accurately due to simplistic strain–stress relation (Vieira et al. 2012; Ammour et al. 2013; Clifford and Kimber 2020). Another disadvantage of the RANS approach is the requirement of complicated stochastic models for particles that are needed to take into account the effect of turbulence. For this sake, LES stands as a common tool for simulating the flow inside 3D cavities. However, to properly resolve the boundary layer, LES has stringent resolution requirements that scale with powers of the Grashof number (Benjamin and François 2017).

The hybrid LES/RANS models emerge as a promising alternative. The RANS-LES approach activates RANS mode near the wall—tolerating much coarser meshes near the wall—while LES is deployed when cell size is sufficient to resolve local motion scales in the far-wall region. In this light, Abramov and Smirnov (2006) studied the square cavity at a Rayleigh number of 1.58*10^9^ using detached eddy simulation (DES) based on the one-equation model for the turbulent kinetic energy (TKE) transport. It was found in this study that although the mean flow was reasonably represented, the flow was observed to be poorly predicted in the near-wall region. Recently, Ali A.E.A (Ali et al. 2021) proposed a dual-mesh hybrid RANS-LES approach to DHC. The idea behind this is to use two overlapping computational domains, one for LES mode and the other to solve RANS equations. The main motivation was to avoid a mismatch between LES and RANS at the interface when a single mesh is used. It was reported that this approach yields a satisfactory accuracy in comparison with standard LES or RANS tested in the same study. The obvious disadvantage is the need to compute both LES and RANS for a single case.

In both works, Abramov and Smirnov (2006), and Ali et al. (2021), periodic boundary condition was imposed in the depth direction, implying an infinitely long domain. Two configurations, with solid boundaries (same set up used in this publication) and with periodic boundaries in the depth direction, produce very different outcomes when the hybrid LES/RANS method is used. The hybrid LES/RANS approach results in a zero modelled energy when applied to the flow in the domain surrounded by solid walls, reducing the simulation to a coarse DNS. This is not a case when the periodicity is imposed in the depth direction. Two configurations produce different turbulent time and length scales and the wall blocking effect is more significant for the domain surrounded by solid boundaries. These differences lead to different performance of the hybrid LES/RANS approach most likely due to different levels of the modelled turbulent kinetic energy production in the near-wall region.

In view of particulate flows in closed cavities, only a few studies have investigated particle tracking in DHC. Some of these studies focused on laminar flow like the work of Akbar et al. (2009) who reported the dispersion of particles of diameter 50 nm to 1 μm. In another study (Bagheri et al. 2012), particle motion was investigated in a 2D DHC cavity flow at a Rayleigh number up to 10^8^. In a turbulent flow regime, both of Puragliesi et al. (2011) and Bosshard et al. (2014) have studied particle transport at Ra = 10^9^ using respectively pseudo-spectral DNS and spectral-element LES codes alongside lagrangian particle tracking (LPT). As a result of the massive CPU requirement, the smallest particle considered in these simulations had a 10 μm diameter—which is relatively large for most of relevant engineering applications.

To the best of our knowledge, the only experimental investigation that provides extensive details for both carrier flow and particle dispersion is the one conducted by Kalilainen et al. (2016). As will be shown later, this experimental data will be used in the exhaustive validation of the primary flow statistics as well as the discrete phase dynamics. Following that study, well-resolved LES was used in the work of Dehbi et al. (2017) to predict particle deposition rates for the range of aerodynamic diameters dp = 0.5–10 μm. In accordance, we use this LES database as a reference for our predictions.

As a continuation of an ongoing project led by the authors where forced flow has been investigated using Wall-Modeled LES (WMLES) and hybrid RANS/LES (Sayed et al. 2020, 2021, 2022), the thermally-driven particulate flow inside a 3D cubical cavity is the focus of the present paper. As URANS and hybrid RANS-LES models fail to reproduce the flow accurately due to difficulty to model subtle physical mechanisms such as laminarization and three-dimensional effects, the alternative is LES applied on a coarse mesh. In this work, the commonly used dynamic Smagorinsky subgrid-scale (SGS) model was employed on a mesh whose resolution is coarser than generally accepted LES standards (hereinafter: Coarse LES (CLES)). The aim is to assess the influence of SGS motions on the main flow statistics and to investigate the feasibility of LES simulations for the particulate flows when a mesh resolution is relatively coarse. In this light, six swarms of different particles sizes were computed once a statistically stationary flow was reached. Point particles are treated as solid spherical elements in a one-way coupling with the primary flow field, where gravity, Stokes drag and thermophoretic forces where considered. In a quantitative fashion, temporally-local particle concentration histories of small-to-medium inertia particles (i.e. 1.4–3.5 μm) were compared to LES and experimental databases. The commonly used simple “stirred settling” model was also employed to compute deposition rates.

The rest of the paper is arranged as follows: in Sect. 2, we show the governing equations. In Sect. 3, we explain the numerical setup and thermal boundary conditions. Results and discussion are shown in Sect. 4 and finally, we give a summary and future recommendations in Sect. 5.

## Governing Equations

### Primary Flow

We consider the momentum, continuity, and energy conservation equations to solve the incompressible Newtonian flow (Eqs. 1–3) with constant viscosity and thermal diffusivity. Density variations due to thermal stratification are accounted for by using the Boussinesq approximation for buoyancy with a constant Prandtl number (0.71). For our CFD simulations, we use the commercial finite-volume CFD code ANSYS Fluent – version 2020 R1 (ANSYS, 2015).

1 $$\frac{{\partial u_{i} }}{\partial t} + u_{j} \frac{{\partial u_{i} }}{{\partial x_{j} }} = - \frac{1}{\rho } \frac{\partial p}{{\partial x_{i} }} + \frac{{\upsilon \partial^{2} u_{i} }}{{\partial x_{i} \partial x_{j} }} + \beta g_{i} \left( {T - T_{ref} } \right) + \frac{{\partial \tau_{ij} }}{{\partial x_{j} }}$$

2 $$\frac{{\partial u_{i} }}{{\partial x_{i} }} = 0$$

3 $$\frac{\partial T}{{\partial t}} + u_{j} \frac{\partial T}{{\partial x_{j} }} = \frac{\partial }{{\partial x_{i} }}\left( {\frac{\upsilon }{Pr} \frac{\partial T}{{\partial x_{i} }} - \underline{\theta u}_{i} } \right)$$

where ${\tau }_{ij}$ is the SGS (residual) shear stress tensor. We use the Linear Eddy Viscosity (LEV) approximation to close the Reynolds stress term that stems from averaging the non-linear advection term in the momentum conservation equation Eq. (1). Using the Boussinesq eddy viscosity assumption (Boussinesq 1877), the stress tensor is evaluated by the space-filtered velocity field, which is linked to the mean velocity gradients. Using this hypothesis, the Reynolds stress tensor reads:

4 $$\tau_{ij} = - \underline{{u_{i} u_{j} }} = \upsilon_{t} \left( {\frac{{\partial u_{i} }}{{\partial x_{j} }} + \frac{{\partial u_{j} }}{{\partial x_{i} }}} \right) - \frac{2}{3}k\delta_{ij}$$

where $k$ is the SGS turbulent kinetic energy (TKE), and ${\delta }_{ij}$ is the Kronecker delta. The subgrid-scale (SGS) eddy viscosity ${\upsilon }_{t}$ is then computed based on the filter width and the Smagorinsky parameter (Eq. 5). Unlike the standard Smagorinsky model, the dynamic one allows Smagorinsky parameter to vary both in space and time depending on an algebraic identity between the subgrid-scale stresses at two different filtered levels and the resolved turbulent stresses (Germano et al. 1991).

5 $$\upsilon_{t} = { }\left( {\Delta_{LES} } \right)^{2} { }C_{dyn} { }\left| S \right|$$

We model the turbulent heat flux in Eq. (3)through the Simple Gradient Diffusion Hypothesis (SGDH) as follows

6 $$\underline{{u_{i} \theta }} = { } - \frac{{\upsilon_{t} }}{{Pr_{t} }}\frac{\partial T}{{\partial x_{i} }}$$

Above ${C}_{dyn}$ is the dynamic Smagorinsky constant, and ${\Delta }_{LES}$ is the standard definition of LES cut-off length (the cubic root of cell volume), and $\left|S\right|= \sqrt{2{S}_{ij}{S}_{ij}}$ is the magnitude of the strain rate tensor (${S}_{ij}$). It will be shown later in Sect. 4 that the Smagorinsky dynamic model can predict the correct eddy viscosity needed to dampen fluctuations in the near-wall region, and hence give a better representation of the flow statistics there.

### Discrete Phase

In this study, particles are treated as point-mass, rigid spheres with perfectly sticking wall collisions. As seen in Eq. (7), each particle is tracked separately and independently in a Lagrangian/Eulerian frame through the particle equation of motion. The advancement of particles is done by interpolating the spatially-filtered fluid velocity at particle location, neglecting the SGS effects on particle motion.

7 $$\frac{{dV_{p, i} }}{dt} = f \frac{{\left( {U_{i} - V_{p,i} } \right)}}{{\tau_{p} }} + g_{i} + F_{th,i}$$

Above ${V}_{p,i}$ is particle velocity in the three orthogonal directions, $f$ is the correction factor for Stokes drag which is computed from:

8 $$f = \frac{{Re_{p} }}{24} C_{d}$$

The drag coefficient ${C}_{d}$ is calculated from

9 $$C_{d} = \beta_{1} + \frac{{ \beta_{2} }}{{Re_{p} }} + \frac{{ \beta_{3} }}{{Re_{p}^{2} }}$$

Above, the ${\beta }_{i}$ are constants which apply for a wide range of spherical particles Reynolds numbers ${Re}_{p}$, and ${g}_{i}$ is the gravitational acceleration vector. The particle response time (also known as particle relaxation time) τ_p_ is defined as:

10 $$\tau_{p} = \frac{{C_{c} \rho_{p} d_{p}^{2} }}{18\mu }$$

${C}_{c}$ is the Cunningham slip-correction factor which is calculated as follows:

11 $$C_{c} = 1 + \frac{\lambda }{{d_{p} }}\left( {2.34 + 1.05 {\text{exp}}\left[ { - 0.39\frac{{d_{p} }}{\lambda }} \right]} \right)$$

To account for temperature effects on particle motion, ${F}_{th,i}$ is the thermophoretic force per unit mass, defined as follows:

12 $$F_{th, i} = - D_{T,p} \frac{1}{{m_{p} T}}\frac{dT}{{dx_{i} }}$$

Above ${m}_{p}$ is particle mass, $T$ is the local temperature at particle location and ${D}_{T,p}$ is the thermophoretic coefficient which is defined as follows:

13 $$D_{T,p} = \frac{{6\pi d_{p} \mu^{2} C_{s} \left( {{\raise0.7ex\hbox{${k_{f} }$} \!\mathord{\left/ {\vphantom {{k_{f} } {k_{p} }}}\right.\kern-\nulldelimiterspace} \!\lower0.7ex\hbox{${k_{p} }$}} + C_{t} k_{n} } \right)}}{{\rho_{f} \left( {1 + 3C_{m} k_{n} } \right)\left( {1 + {\raise0.7ex\hbox{${2k_{f} }$} \!\mathord{\left/ {\vphantom {{2k_{f} } {k_{p} }}}\right.\kern-\nulldelimiterspace} \!\lower0.7ex\hbox{${k_{p} }$}} + 2C_{t} k_{n} } \right)}}$$

Above ${C}_{s}$, ${C}_{t}$, ${C}_{m}$ are the dimensionless constants of the Talbot model (Talbot et al. 1980) having the values of 1.17, 2.18 and 1.14 and ${k}_{f}$ and ${k}_{p}$ are the fluid and particle thermal conductivities taking the values 2.434E-5 and 0.02723 W m^−1^ k^−1^ respectively. The Knudsen number ${k}_{n}$, which is defined as the ratio of the medium mean free path to the characteristic length of the considered system reads:

14 $$k_{n} = \frac{2\lambda }{{d_{p} }}$$

$\lambda$ is the air mean free path (i.e. the average distance atoms or molecules travel between successive collisions) having the value 0.066 µm at 101 kPa and 293 K, and ${d}_{p}$ is the particle diameter. Monodispersed silica (SiO_2_) particles with Aerodynamic Mean Mass Diameter (AMMD) of 1.4 – 14 µm were considered where actual particle density is 2000 kg/m^3^. Once particle velocity is computed, particle local position can be calculated from the following equation:

15 $$\frac{{dx_{p, i} }}{dt} = V_{p,i}$$

For time integration, we use Runge–Kutta fourth-order (RK4) scheme, while spatial interpolation of fluid velocity at particle location is done by three-dimensional linear interpolation. To have a direct comparison with the experimental and LES databases of both Kalilainen et al. ( 2016) and Dehbi et al. (2017), other forces (e.g. lift, turbulent dispersion, Brownian diffusion, etc.) are not accounted for in our simulations. Particle tracking is done in a post-processing step after computing the primary flow at each fluid time step.

## Numerical Setup and Thermal Boundary Conditions

We consider a cubical cavity (x: horizontal, y: depth, z: vertical) with a side length of 0.7 m, where all walls are set to the no-slip boundary condition. Regarding thermal boundary, the flow inside the cavity is buoyancy-driven whereby the vertical walls are held at two different temperatures with ΔT = 39.18 to have a Rayleigh number of Ra = 10^9^. As in the fine LES study by Dehbi et al. (2017), the front and back walls were set to adiabatic (passive) walls in all studied cases. Reference temperature (${T}_{ref}= \frac{{T}_{H}-{T}_{C}}{2}$) and was set to 310.95 K. For turbulent heat flux calculation, the standard Fluent value of the turbulent Prandtl number (${Pr}_{t}$) was used i.e. 0.85. It is worth mentioning that the choice of such value is not very straightforward. This is due to its dependence on kinetic energy and scalar spectral transfers, which makes ${Pr}_{t}$ case-specific, and therefore quite challenging to assign a value to it. For instance, the value 0.4 is often used in LES as it was shown that this value dominates in the far-from-wall region (where turbulence is fairly isotropic). However, this value can increase to reach approximately 1.0 for air near the wall as reported by Kim and Moin (1989), who admittedly considered a forced convection case. Such higher values were also reported in Gibbs and Fedorovich (2016) who used values between 0.4 and 1.0 based on the defined criterion that takes the stability-dependent length-scale into account. In addition, it was reported by Moin et al. (1991) that the coarser the employed grid, the more likely it is for the turbulent Prandtl number to move toward the typical value used in RANS. Since we use a relatively coarse grid, we adopted the standard Fluent value of ${Pr}_{t}$. Fortunately, this value is not expected to influence temperature statistics much, and therefore, the choice of ${Pr}_{t}$ value is not very critical in our case.

As reported in the literature, it is crucial to account for wall-to-wall radiation (between the bottom and top walls) (Ali et al. 2021; Ibrahim et al. 2013; Sergent et al. 2013a, 2013b; Xin et al. 2013). We therefore implicitly account for such effect by imposing the measured temperature profiles from the experiment as Dirichlet boundary conditions as suggested by Dehbi et al. (2017). It should be pointed out that the authors in Dehbi et al. (2017) used the dynamic kinetic energy SGS model. The model is very similar to the dynamic Smagorinsky model, but it solves one additional equation to explicitly quantify the SGS TKE. As will be shown in the results section, the dynamic model with TKE transport yields similar results. Therefore, the dynamic Smagorinksy model can be sufficient in this case for an efficient particle tracking analysis.

In a quantitative fashion, we report flow first and second-order statistics i.e. mean and RMS velocity profiles as well as mean temperature profiles at different locations across the cavity. Results are compared against reference LES predictions by Dehbi et al. (2017) and experimental measurements by Kalilainen et al. (2016). In particular, the high-quality particle image velocimetry (PIV) experimental measurements produced by Kalilainen made it possible to quantitatively validate our results. In addition, we qualitatively show the main characteristics of the flow circulation at the cavity corners—where the flow is turbulent, as well as other flow coherent structures.

**Fig. 1:**
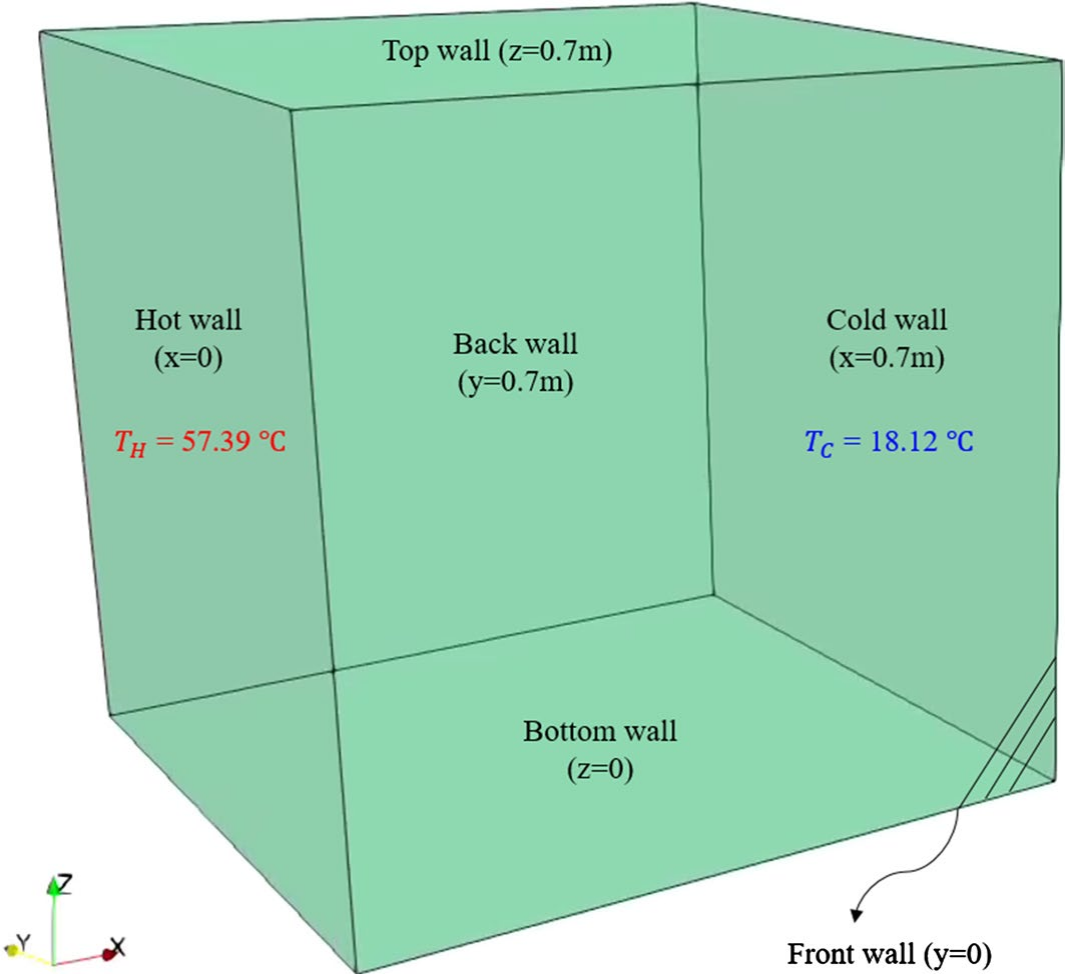
Schematic showing the geometry of the cavity with the thermal boundary conditions indicated

**Fig. 2:**
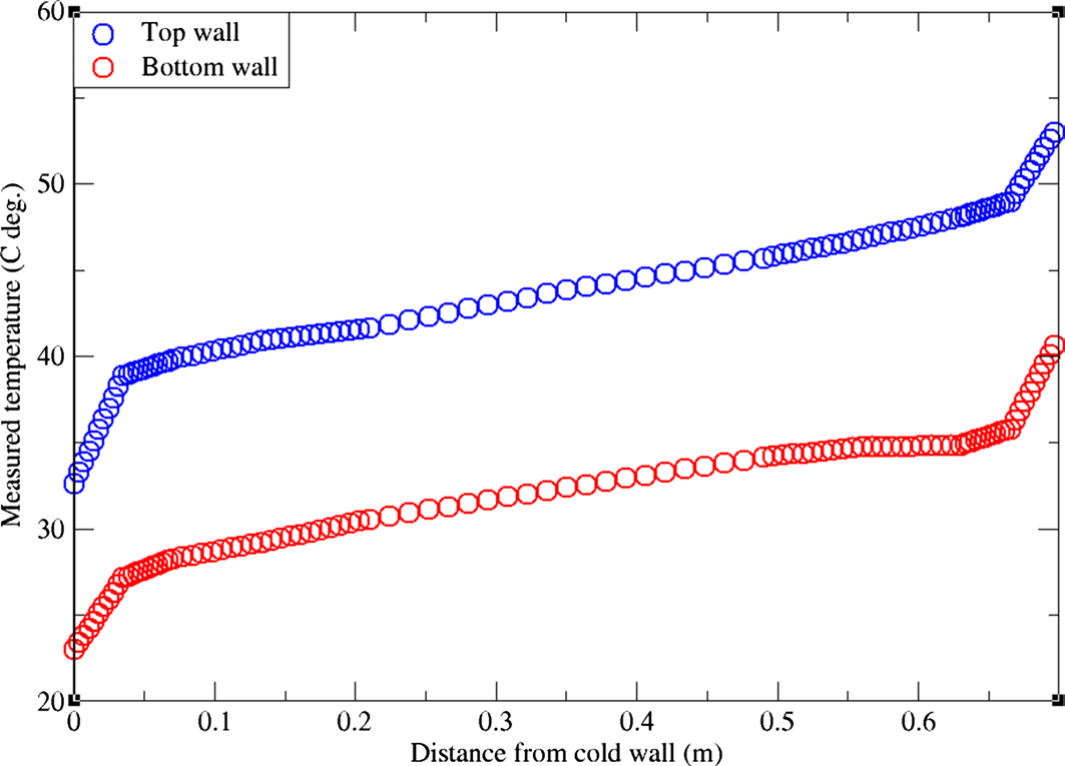
Measured temperature profiles for bottom and top walls from exp. Kalilainen et al. (2016)

As schematized in Fig. 1, the left vertical wall (x = 0) is assigned to be the hot wall (at temperature T = 330.54 K), while the cold wall is at x = 0.7 (with T = 291.36 K). It was mentioned by Kalilainen et al. (2016) that the isothermal wall temperature measurements in DIANA (DIfferentially heated cavity with Aerosol in turbulent NAtural convection) experiment had a 0.4 K uncertainty. Also, due care was taken to reduce heat losses from passive walls to the surroundings so that the adiabatic wall assumption is reasonable. To implicitly emulate wall-to-wall radiation effects, we set the temperature of bottom and top walls independently of the spanwise direction (y-axis). This is done by imposing the measured temperature profiles (from the DIANA experiment) as Dirichlet boundary conditions on the horizontal walls as in Fig. 2. This set of boundary conditions are called Intermediate Realistic Conditions (IRC) as first coined by Xin et al. (2013). In that study, the authors showed that IRCs can represent the right physics of the turbulent flow field in the DHC. It should be pointed out that, due to imperfections in the experimental measurements, the measured temperature profiles on both top and bottom walls are not perfectly symmetrical (as in Fig. 2). Therefore, the obtained velocity profiles by the simulation are not expected to be symmetrical in any of the reported quantities. This will be seen later in the results section.

As indicated in Table 1, the fluid parameters used is air with the physical properties fixed for all simulations. For pressure momentum coupling, we use the SIMPLE scheme and for time integration, we use the second order scheme. The bounded central differencing (BCD) scheme was used for the momentum equation while the second order upwind scheme was used for the energy equation. This is the recommended and default setup by ANSYS Fluent as it provides the least dissipative solution providing the highest resolution accuracy for the smallest scales. The turbulent heat flux model has been set to the simple gradient diffusion hypothesis (SGDH) as in Eq. (7).

**Table 1:** Input values for fluid parameters

Variable	$\rho$[kg/m^3^]	${k}_{f}$[W/(m⋅K)]	β [1/K]	υ [m^2^/s]	${c}_{p}$[KJ/kg.K]
Values	1.0	2.434E-5	0.0313	1.728E-5	1.0

Once a statistically-stationary flow is reached, particles are initially distributed in random positions covering the whole domain, and released with zero velocity. Since we are aiming at the collective behavior of particle dispersion, we track particles under simplified conditions in which particle size and concentration are small enough to assume very dilute flow. This implies that particle–particle collision is neglected, and that particle has no feedback on the carrier fluid. Six swarms covering the range of particle aerodynamic diameters 1.4–14 µm were considered. To compute particle removal rates, all walls were assumed to be perfectly absorbing. Once the particle center of mass is past the wall closest cell center, it is considered deposited and removed automatically from the domain. Particle inertia is usually characterized by Stokes number which is defined as the ratio of particle relaxation time to a fluid time scale:

16 $$St = \frac{{\tau_{p} }}{{\tau_{f} }}$$

In this flow configuration, the time unit is defined in terms of cavity length and circulation speed as follows:

17 $$\tau_{f} = \frac{L }{{V_{r} }}$$

18 $$V_{r } = \frac{{\alpha \sqrt {Ra} }}{L}$$

To achieve time-accurate solution for particle motion, the particle time step (Δt_p_) must be of the same order of magnitude as the particle time scale ($\le {\tau }_{p}$/2). For this sake, the code calculates the needed number of sub time steps assigned for each particle size so that it corresponds to the flow (global) time step. As a result, particle time step for sub-micro particles becomes very tiny, and hence, only few time realizations could be obtained for smaller particles. The time integration was performed by the so-called automatic tracking scheme algorithm which, for computational efficiency alternates between the first order implicit Euler scheme to the second order Trapezoidal scheme, depending on the local fluid dynamics. An embedded error function tracing particle trajectory is enforced, such that particle time step is sufficiently reduced until the predicted error in the trajectory drops below a certain tolerance. To guarantee a time-accurate solution for particles, this tolerance is set to a very low value of 1E-7 m, which is 100 times smaller than the smallest particle diameters considered (1.4 μm) as shown in Table 2.

**Table 2:** Particle parameters

d_p_ [$\mu$ m]	d_AMMD_ [$\mu$ m]	C_c_ [-]	${\tau }_{p}$(E-5) [s]	St (E-5) [-]
1.0	1.4	1.155	0.74	1.21
2.5	3.54	1.062	4.27	6.94
3.5	5.0	1.044	8.22	13.37
6.0	8.49	1.026	23.74	38.61
8.0	11.31	1.019	41.95	68.21
10.0	14.14	1.015	65.29	106.17

## Results and Discussion

### Eulerian Statistics

In this section, we show and discuss the results from pure LES with the dynamic Smagorinsky SGS model. It is usual to judge the predictions of the flow statistics collected after a certain number of flow time units, ${\tau }_{f}$.

In the LES study of Puragliesi et al. (2010), authors consider 400 time units to assume statistically stationarity (fully developed flow), while both studies by Dehbi et al. (2017) and Sergent et al. (2013a) quote 600 time units. In this study, we adopt double the latter timespan, where turbulent statistics were initiated after 1200 time units from the beginning of the simulation, then results were averaged over another 1200 time units. This time-averaging interval was found to ensure full representation of the flow realizations. For our simulations, the circulation velocity and the time unit have values of 1.138 m/s and 0.615 s respectively. Considering the time step size of 0.01 s, this translates into approximately 150,000 time steps for the whole simulation.

**Fig. 3:**
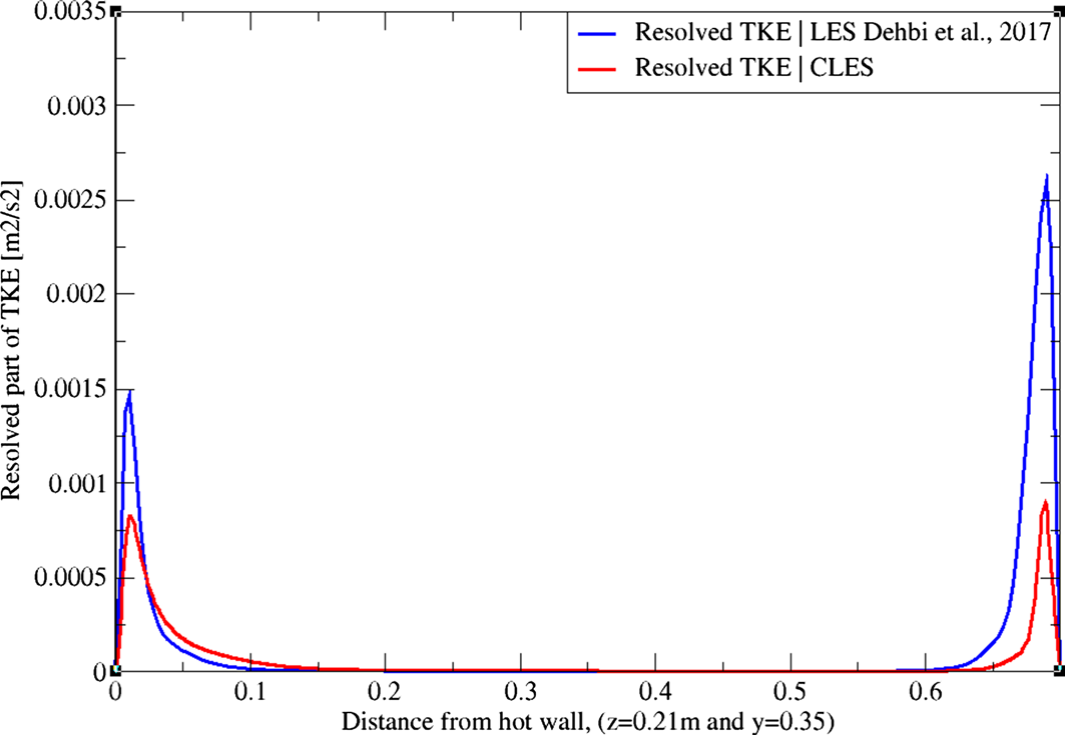
TKE profiles from Dehbi et al. (2017) (blue) and CLES dynamic (red). Profiles are reported across the cavity height (y = 0.21 m)

**Fig. 4:**
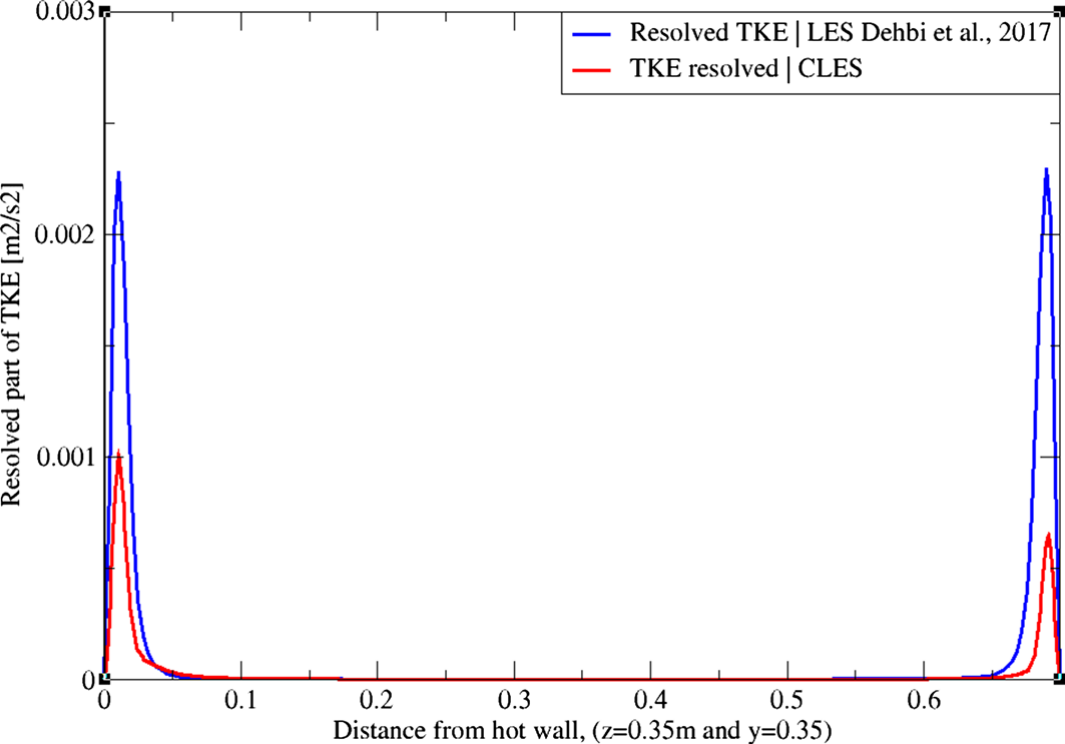
TKE profiles from Dehbi et al. (2017) (blue) and CLES dynamic (red). Profiles are reported across the cavity height (y = 0.35 m)

**Fig. 5:**
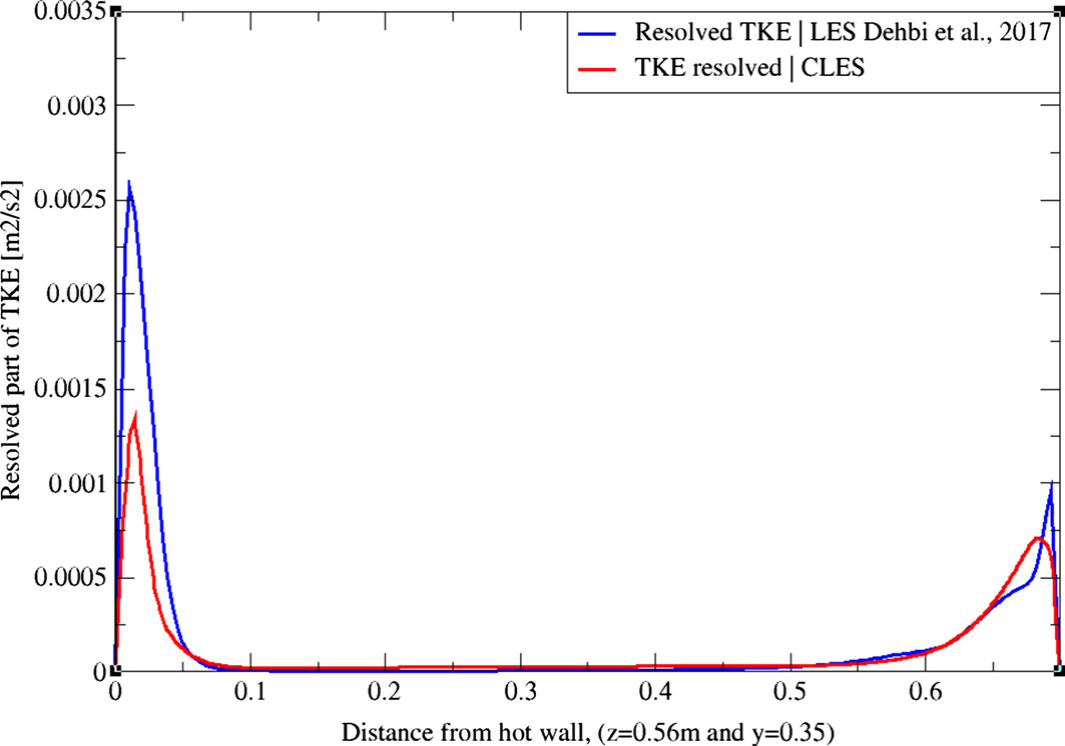
TKE profiles from Dehbi et al. (2017) (blue) and CLES dynamic (red). Profiles are reported across the cavity height (y = 0.56 m)

Using the LES data from Dehbi et al. (2017) as a reference, we assess our results by comparing the resolved part of TKE at three different sections across the cavity height as in Figs. 3, 4, and 5. It can be noticed that at the mid-plane (z = 0.35 m and y = 0.35 m) the dynamic Smagorinsky model is capable of capturing only about 50% of the TKE in the near-wall region. It can also be noticed that the SGS effects are more pronounced close to the top and bottom corners of the cavity where thermal stratification is prominent.

LES is a transient approach whose solution depends on the grid employed. For this reason, one should only seek grid sensitivity in terms of turbulent statistics predictions. As mentioned by Pope (2000), a mesh that captures 80% of the total turbulent kinetic energy (TKE) is deemed sufficient for a well-resolved LES. It was shown in Dehbi et al. (2017) that a 2.4 million-cell mesh (in comparison with 5.1 million-cell mesh) is sufficient for producing a well-resolved LES. As mentioned above, we use relatively coarse grids to solve the flow.

The number of elements used across each edge in the fine LES is 125 with a growth rate of 1.2 and a bias factor of 1.05. As shown in Table 3 below, a coarser grid of 50 elements was refined near the walls with a stretching factor of 1.2 such that the corresponding y^+^ values for both hot and cold walls (where the velocity magnitude is biggest) are below 1. To guarantee a time-accurate solution, the time step size was assigned in each case so that the maximum Courant–Friedrichs–Lewy number (CFL) is less than one throughout the simulation in all reported cases. Since the aim is always to place the first computational cell in the viscous sublayer (i.e. y^+^ < 3), the mesh coarseness can be judged by the maximum cell size, $\mathrm{max}(\Delta {X}_{i})$. This happens to be in the bulk region in this flow configuration. In our case, the coarse mesh has a ${\text{max}}\left( {\Delta X_{i} } \right)$ of 0.055 H compared to 0.014 H in Dehbi et al. (2017).

**Table 3:** Meshes used for all simulations

N_x_	N_y_	N_Z_	N_tot_	${y}_{1}^{+}$)_cold_	${y}_{1}^{+}$)_hot_
50	50	50	125,000	0.87	0.86

**Fig. 6:**
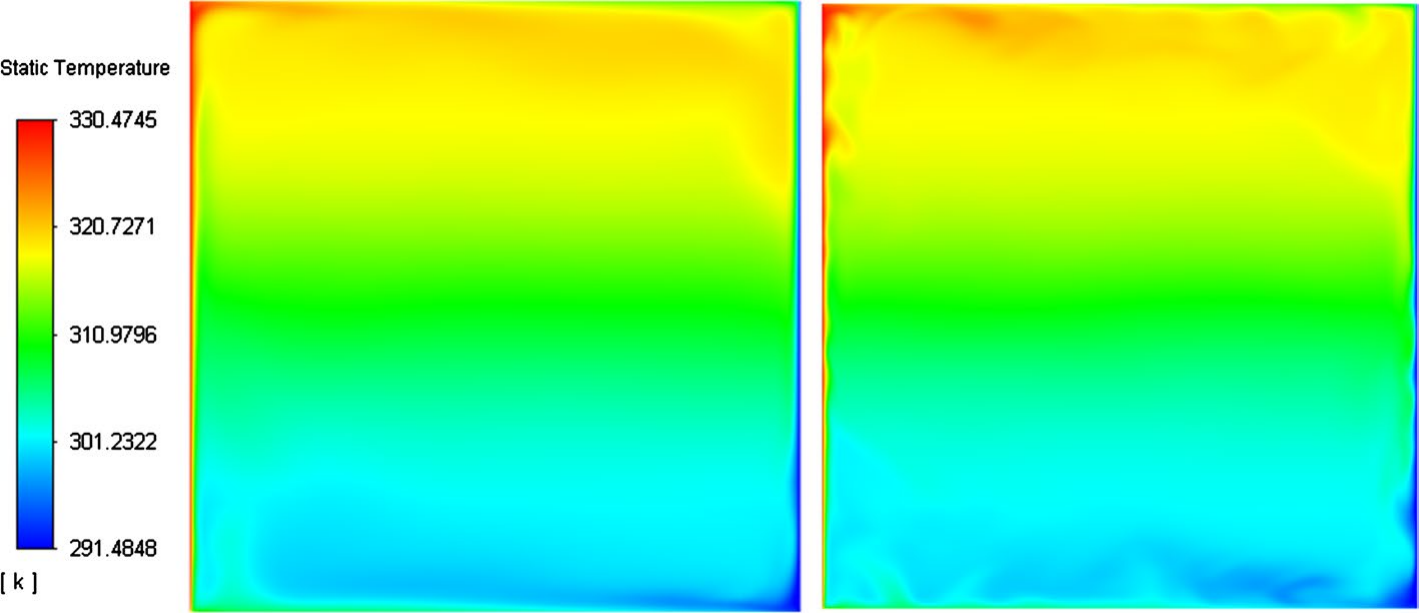
Instantaneous temperature field from well-resolved LES from Dehbi et al., 2017 (right) and own CLES dynamic results (left) at the cavity mid-plane (x = 0.35 m) by the Smagorinsky dynamic model after 2400 τ

**Fig. 7:**
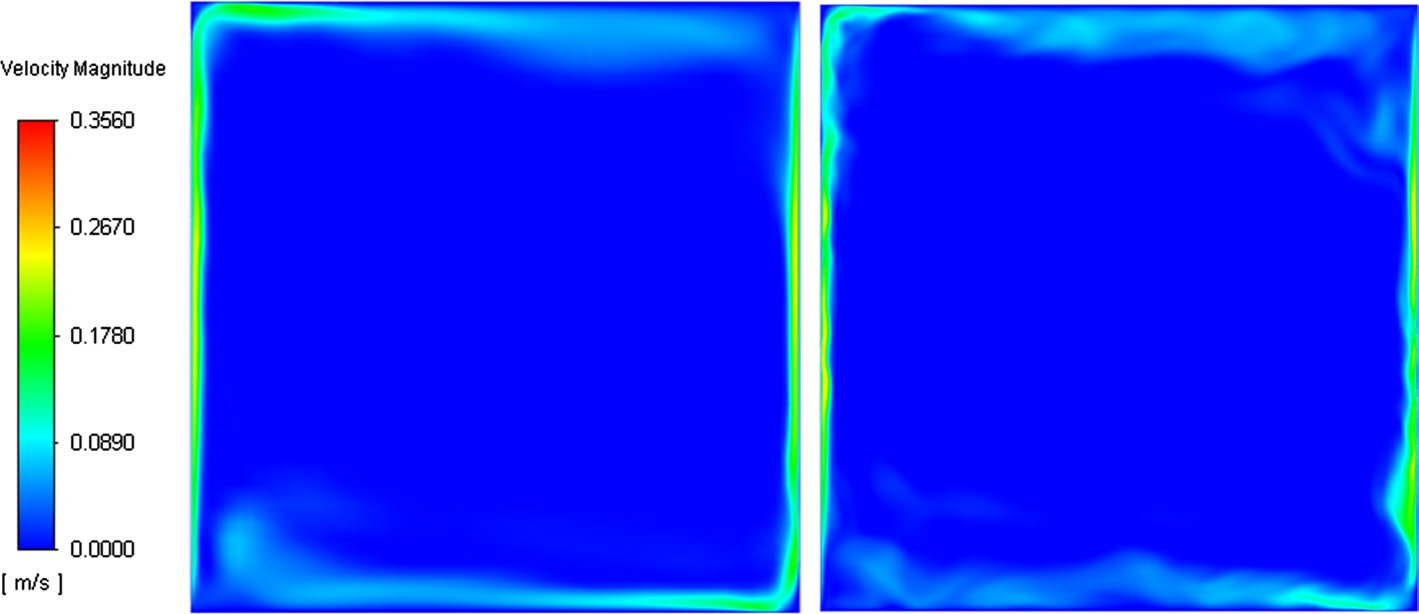
Instantaneous velocity magnitude from well-resolved LES from Dehbi et al., 2017 (right) and own CLES dynamic results (left) at the cavity mid-plane (x = 0.35 m) by the Smagorinsky dynamic model after 2400 τ

The DHC has a unique flow configuration with two distinctive thermal and velocity boundary layers developing on the vertical active walls. The thickness of these boundary layers scales with ${Ra}^{-1/4}$ (Puragliesi 2011). This special flow regime can be observed from both the temperature and velocity contours (Figs. 6 and 7) where the highest velocity is located near the active hot and cold walls, while in the bulk region the fluid velocity almost nullifies. This can also be observed from the velocity contours shown in Fig. 7, where two main counter-currents co-exist at the outer region of the vertical boundary layers. The reason for this particular flow constellation is due to the buoyancy force emerging from thermal stratification (see Fig. 6). In addition, the centrosymmetric property can be identified from the velocity and temperature fields (Figs. 6 and 7). It is important to highlight that the turbulent flow structures are more dampened in our flow field representation compared to the reference LES from Dehbi et al. (2017). This is mainly due to the relatively coarse mesh employed which accounts for bigger eddies according to the cut-off length.

**Fig. 8:**
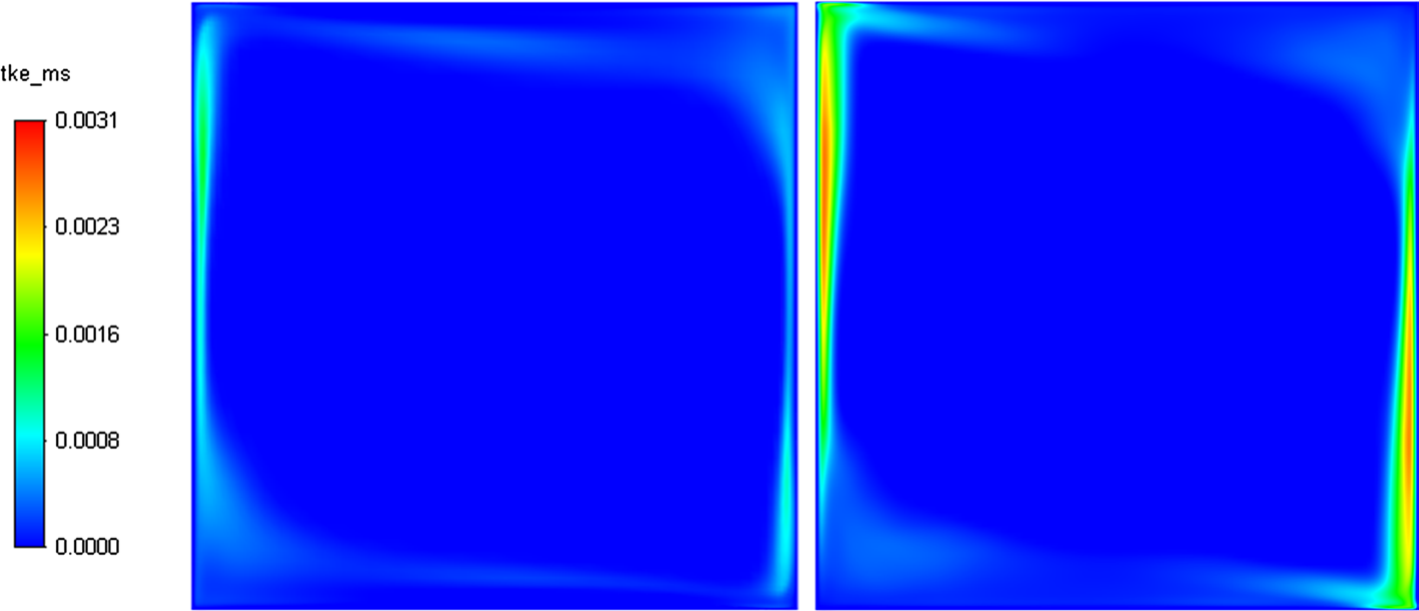
Resolved part of turbulent kinetic energy at the mid-plane (y = 0.35) from both reference LES by Dehbi et al., 2017 (right), and own CLES dynamic results (left)

On another note, it can be observed qualitatively from Fig. 8 that the TKE is underpredicted by the dynamic Smagorinsky model on the relatively coarse mesh employed. It should also be mentioned that velocity gradients are higher at the cavity corners due to the strong flow recirculation pockets. This particular flow regime makes the choice of the sub-grid scale model for wall treatment quite crucial. For this sake, we study with scrutiny the capability of the dynamic Smagorinsky SGS model to predict the turbulent statistics in this benchmark.

In addition to the above qualitative assessment, a quantitative analysis of flow mean and root mean square (RMS) profiles is reported for temperature and velocity fields. Profiles are plotted for the dynamic model to reveal the effect of the SGS model on flow prediction in the near-wall region—which is our main interest for the next step of particulate cavity investigation. To have a one-to-one comparison with reference LES and experimental databases, flow statistics have been reported at four different locations across the cavity (x = 0.35, 0.56 m between the horizontal walls, and Z = 0.35, 0.56 m between vertical active walls).

**Fig. 9:**
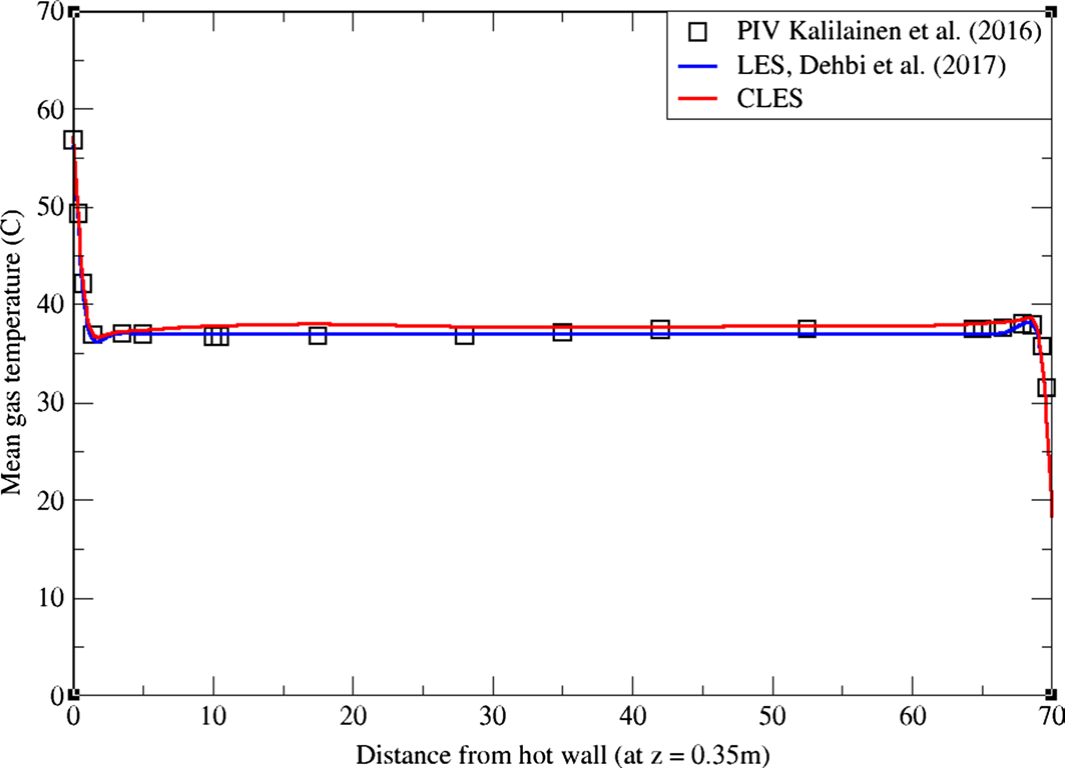
Comparison for mean temperature profiles between hot and cold walls from the dynamic Smagorinsky model against reference LES and experimental databases. Profiles are obtained at (z = 0.35 m) after 2400 time units

**Fig. 10:**
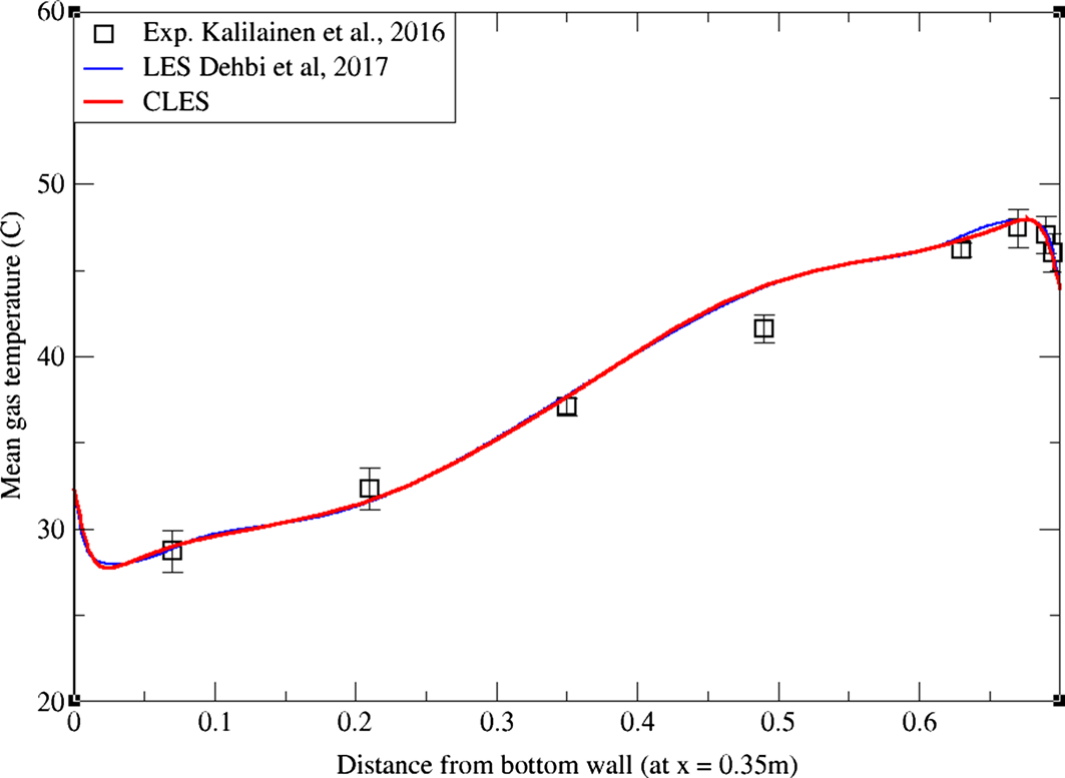
Comparison for mean temperature profiles between bottom and top walls from the dynamic Smagorinsky model against reference LES and experimental databases. Profiles are obtained at (x = 0.35 m) after 2400 time units

**Fig. 11:**
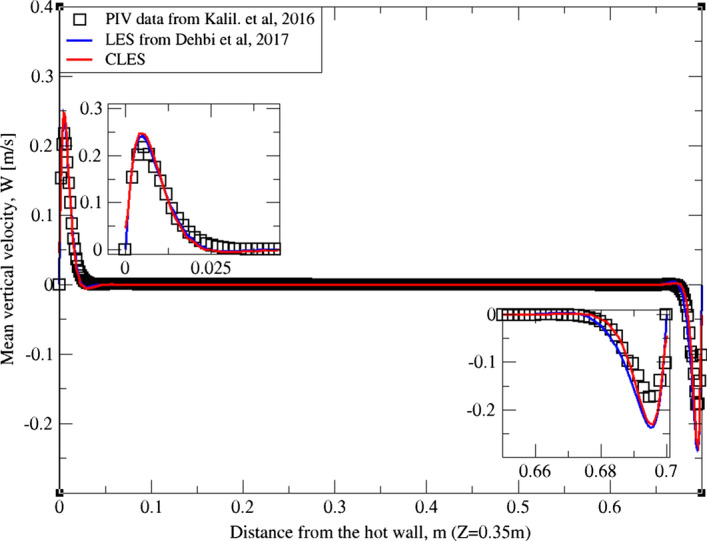
Comparison for mean vertical velocity profiles between hot and cold walls from the dynamic Smagorinsky model against reference LES and experimental databases. Profiles are obtained at (z = 0.35 m) after 2400 time units

**Fig. 12:**
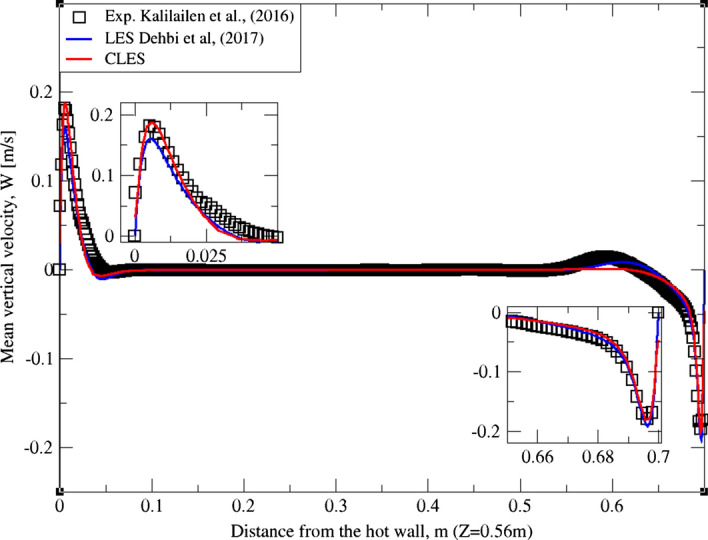
Comparison for mean vertical velocity profiles between hot and cold walls from the dynamic Smagorinsky model against reference LES and experimental databases. Profiles are obtained at (z = 0.56 m) after 2400 time units

**Fig. 13:**
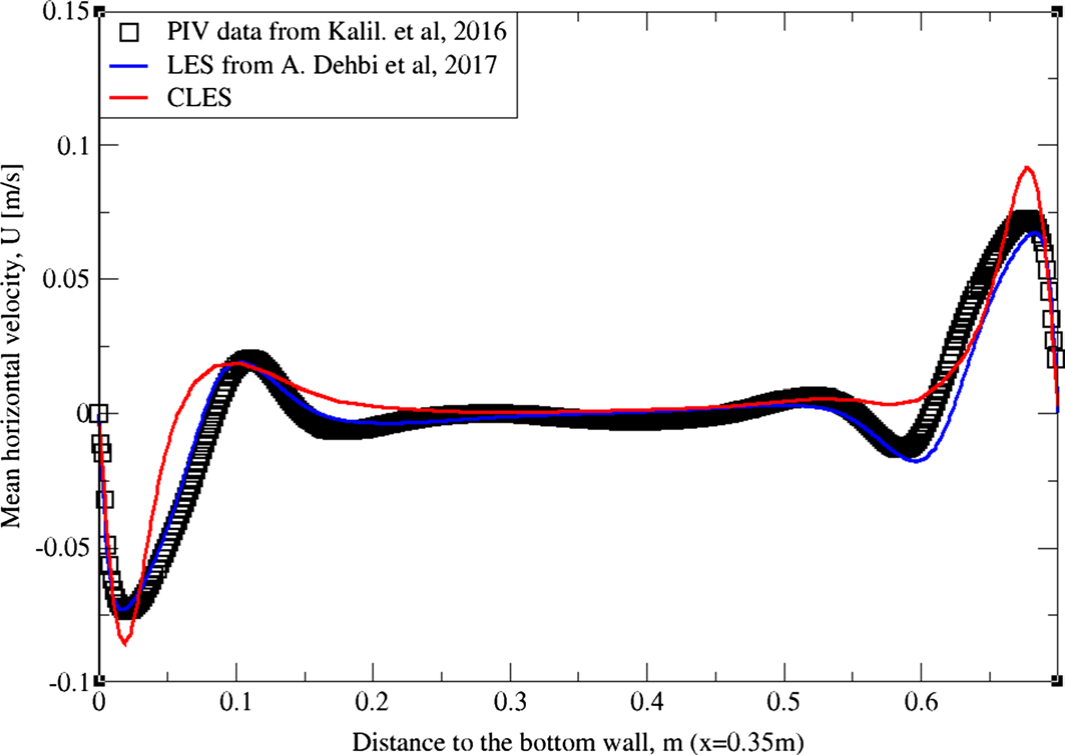
Comparison for mean horizontal profiles between bottom and top walls from the dynamic Smagorinsky model against reference LES and experimental databases. Profiles are obtained at (x = 0.35 m) after 2400 time units

**Fig. 14:**
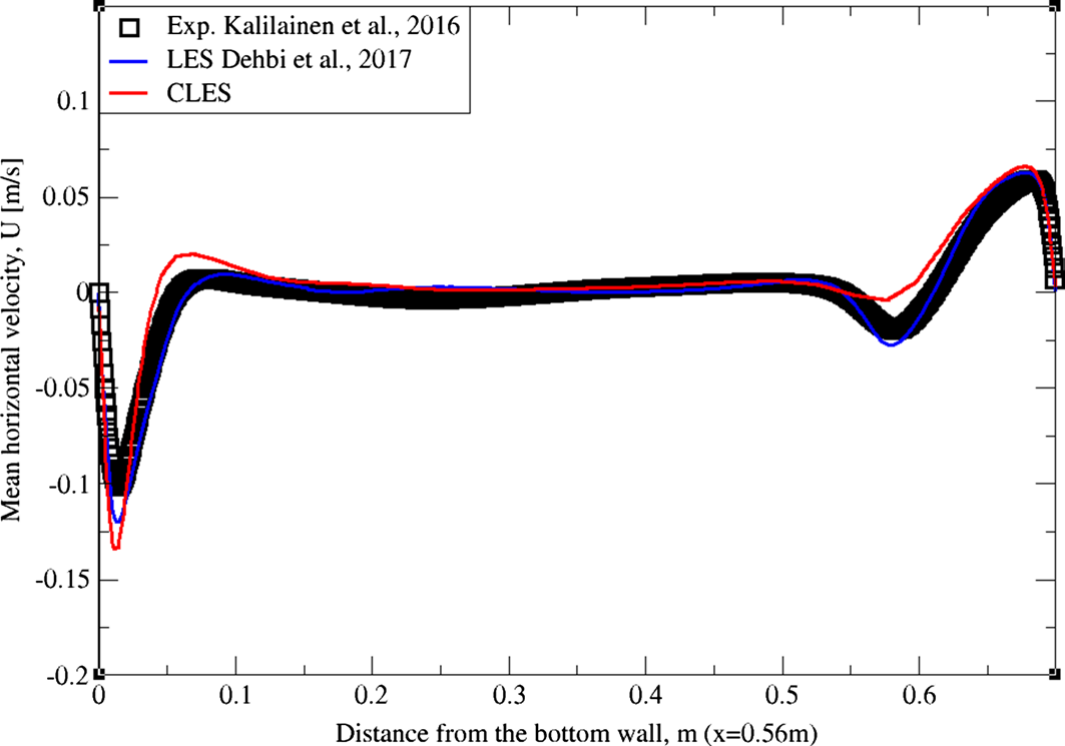
Comparison for mean horizontal profiles between bottom and top walls from the dynamic Smagorinsky model against reference LES and experimental databases. Profiles are obtained at (x = 0.56 m) after 2400 time units

As can be noticed from Figs. 9, 10, 11, 12, 13, and 14, the mean temperature and velocity profiles for both the horizontal and vertical centerlines (x = 0.35 m and z = 0.35 m) give a very good match with the reference data. However, for RMS velocity profiles, the SGS model effect is more pronounced. It must be noted that mean temperature profiles are not very sensitive to the grid resolution as it is the case for mean velocity field. This can seen from Figs. 9 and 10 where our CLES predictions match the reference LES and experimental data. On the other hand, the predicted mean velocity profiles especially for the horizontal component (Figs. 13 and 14) show some deviation from the reference data at both planes i.e. x = 0.35 m, 0.56 m. It is not surprising that such profiles are slightly out of phase in the bulk region due to insufficient mesh resolution. Conversely, the mean vertical component shows an excellent agreement with reference data at both measuring locations (Figs. 11 and 12).

**Fig. 15:**
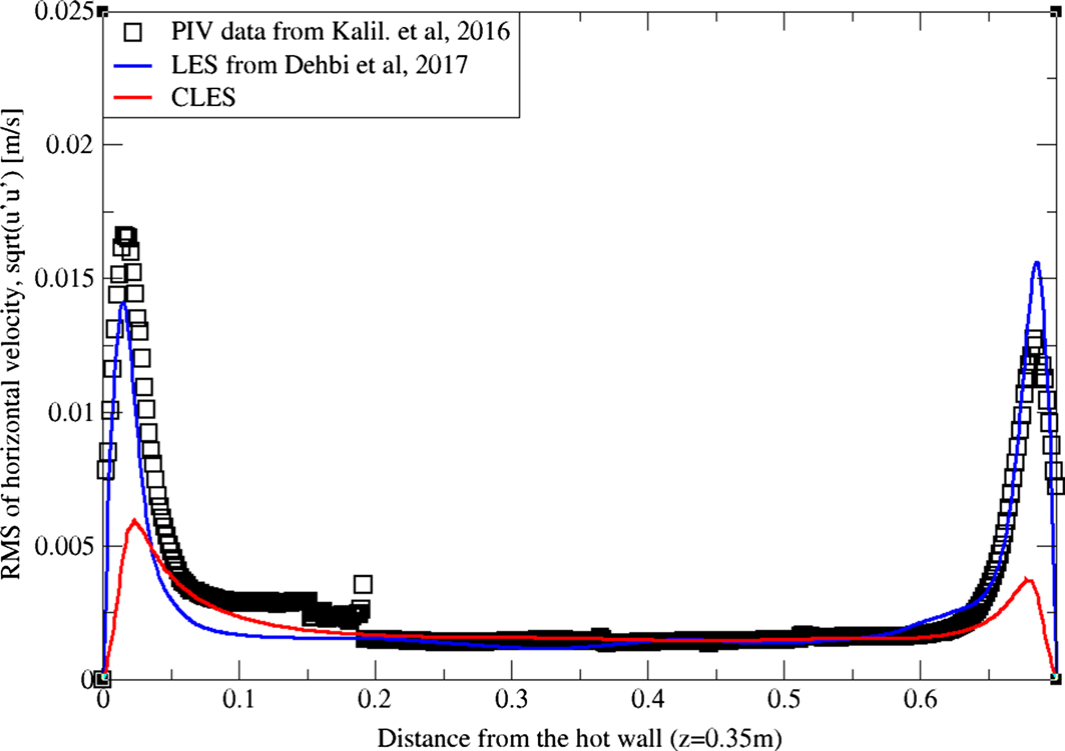
Comparison for RMS horizontal velocity between hot and cold walls from the dynamic Smagorinsky model against reference LES and experimental databases. Profiles are obtained at (z = 0.35 m) after 2400 time units

**Fig. 16:**
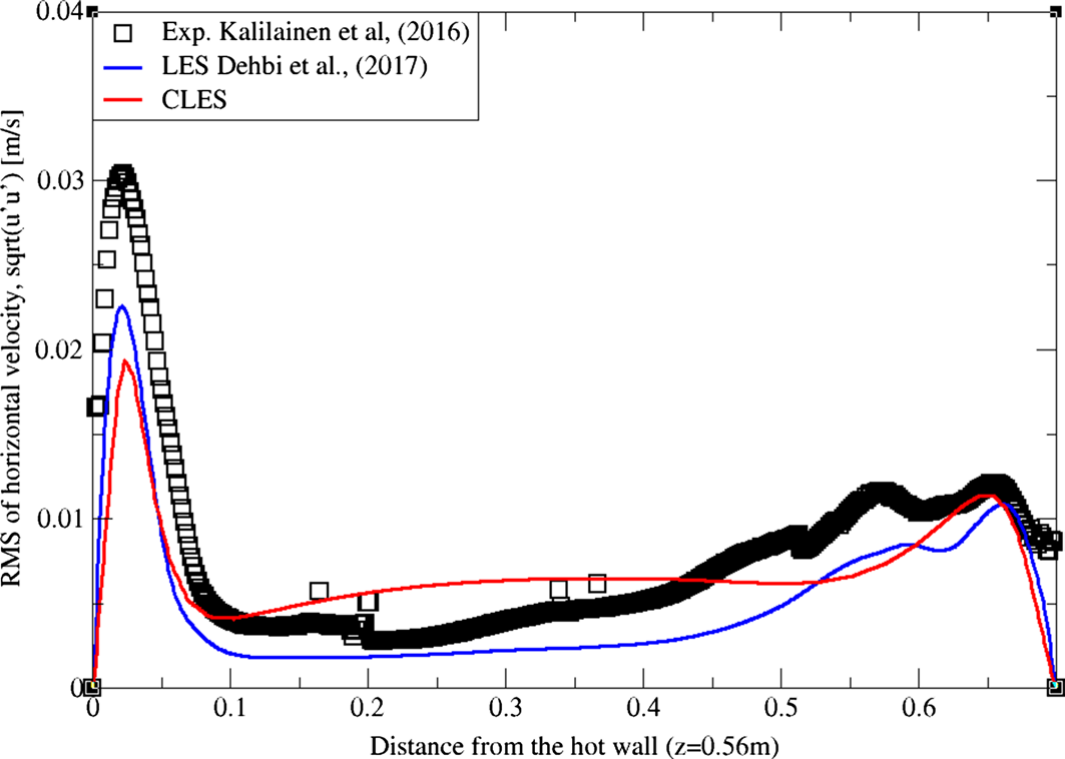
Comparison for RMS horizontal velocity between hot and cold walls from the dynamic Smagorinsky model against reference LES and experimental databases. Profiles are obtained at (z = 0.56 m) after 2400 time units

**Fig. 17:**
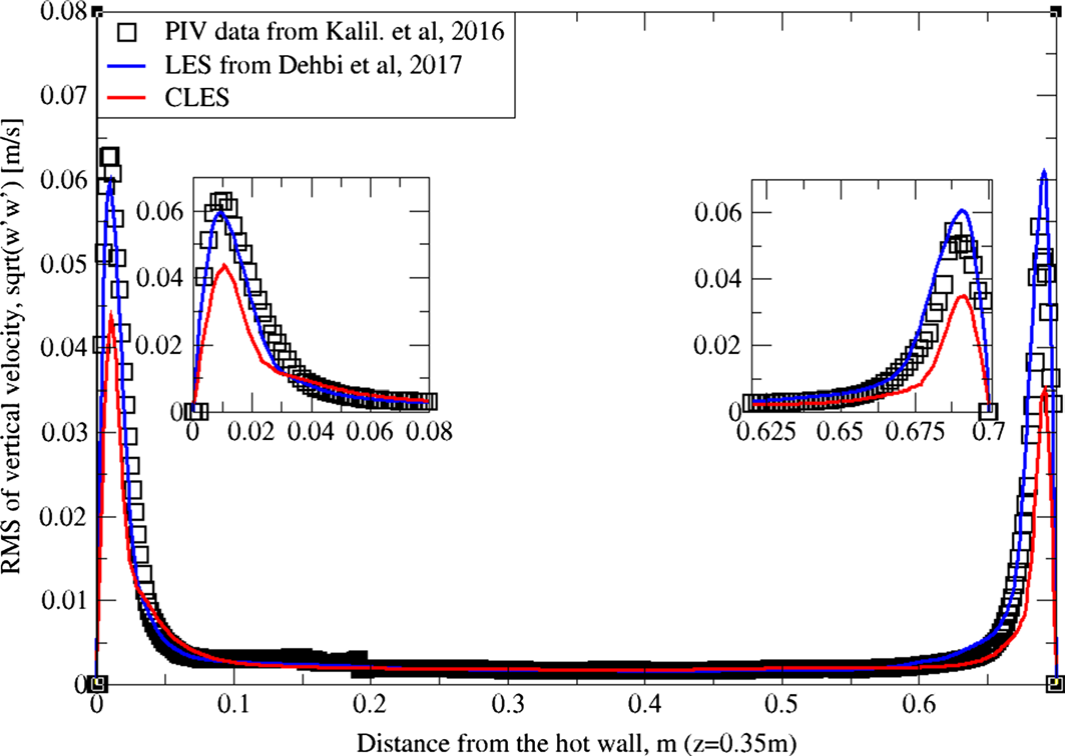
Comparison for RMS vertical velocity between hot and cold walls from the dynamic Smagorinsky model against reference LES and experimental databases. Profiles are obtained at (z = 0.35 m) after 2400 time units

**Fig. 18:**
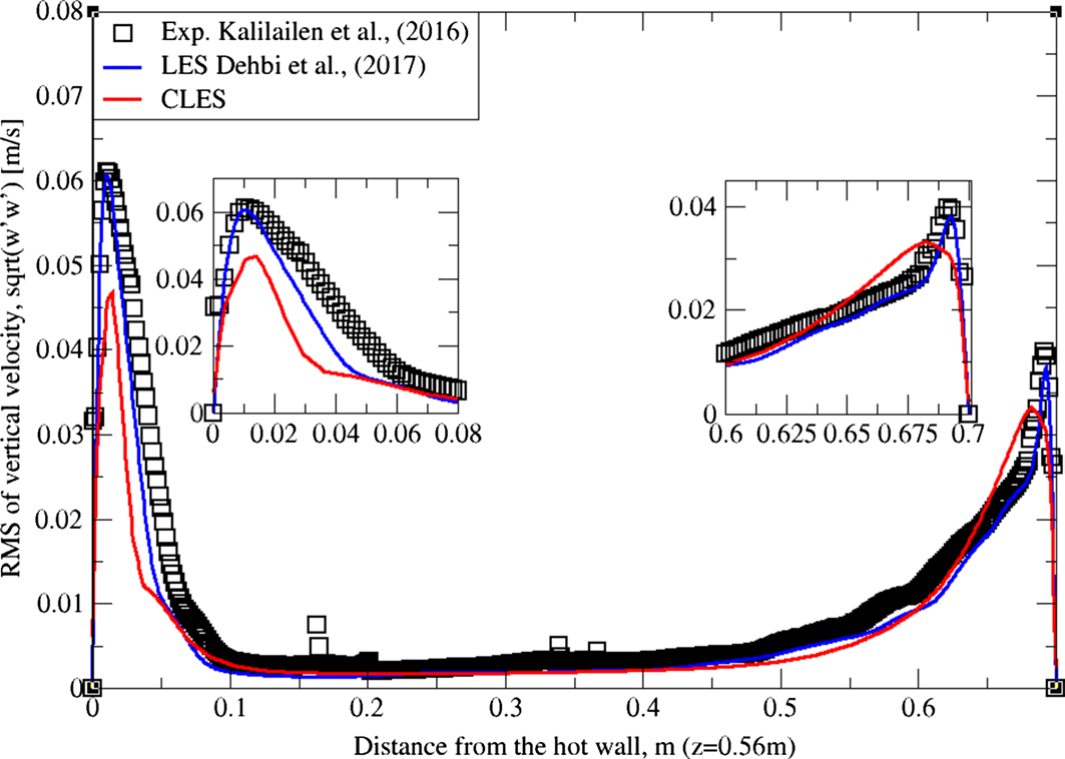
Comparison for RMS vertical velocity between hot and cold walls from the dynamic Smagorinsky model against reference LES and experimental databases. Profiles are obtained at (z = 0.56 m) after 2400 time units

Similarly, for higher moment statistics we report velocity fluctuations i.e. RMS values of both horizontal and vertical velocities (Figs. 15, 16, 17 and 18). The dynamic model in contrast is shown to considerably underpredict the RMS of the horizontal velocity component (Figs. 15 and 16). However, it shows a better match with the vertical component. This can be seen in RMS profiles at z = 0.35 m (Fig. 17) where the dynamic model captures 73% of the peak value of the velocity fluctuations, which is a sufficiently good prediction for a further particle tracking. Closer to the top wall (z = 0.56 m), the dynamic model still captures around 75% of the peak values at the wall relative to the reference LES (Fig. 18).

**Fig. 19:**
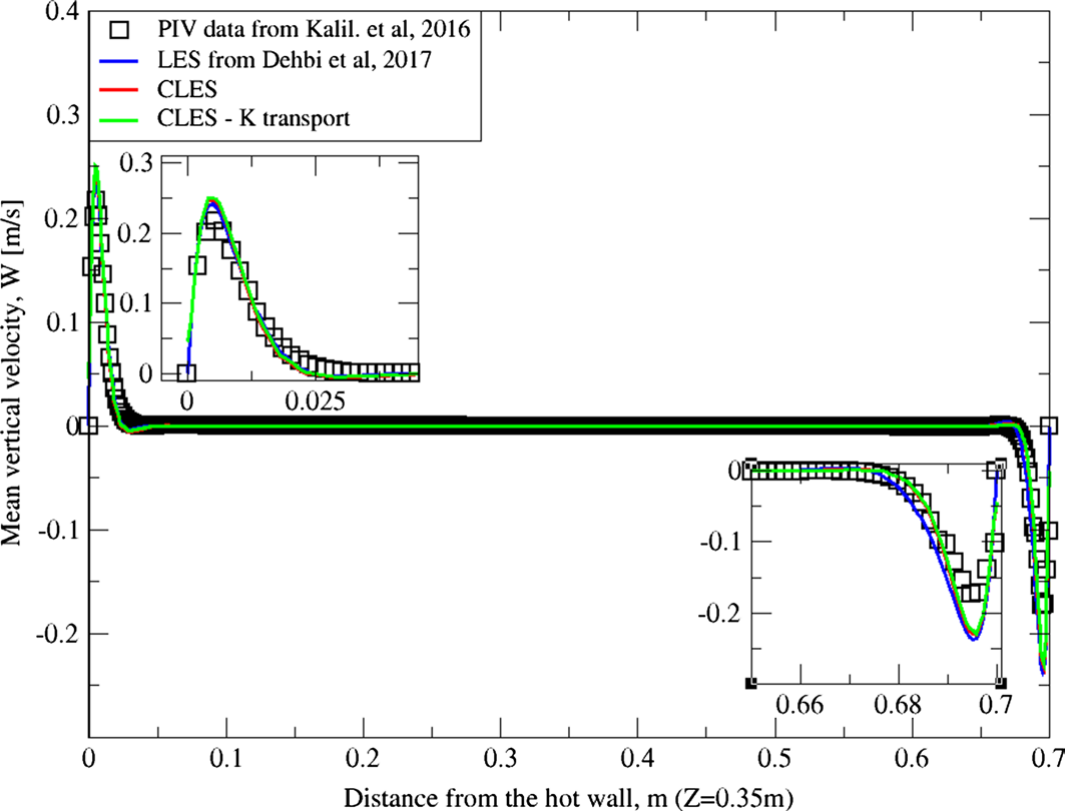
Comparison for mean vertical velocity profiles between hot and cold walls (at z = 0.35 m) from both the standard dynamic Smagorinsky model and the dynamic model with TKE transport against reference LES and experimental databases

**Fig. 20:**
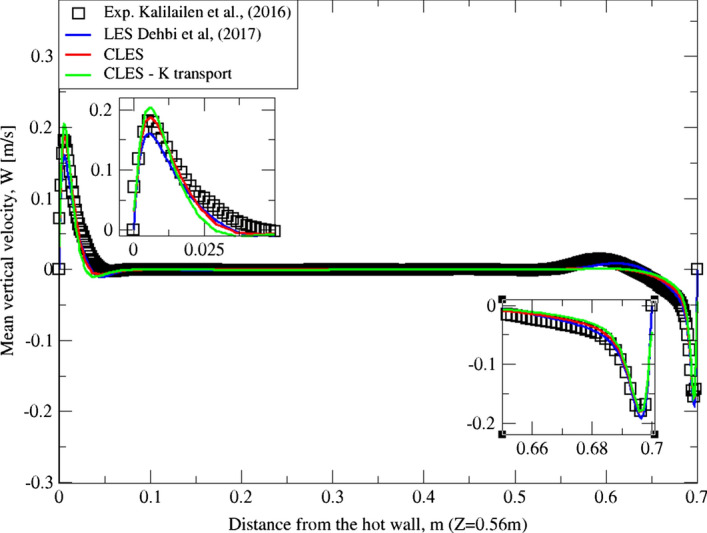
Comparison for RMS vertical velocity profiles between hot and cold walls (at z = 0.56 m) from both the standard dynamic Smagorinsky model and the dynamic model with TKE transport against reference LES and experimental databases

**Fig. 21:**
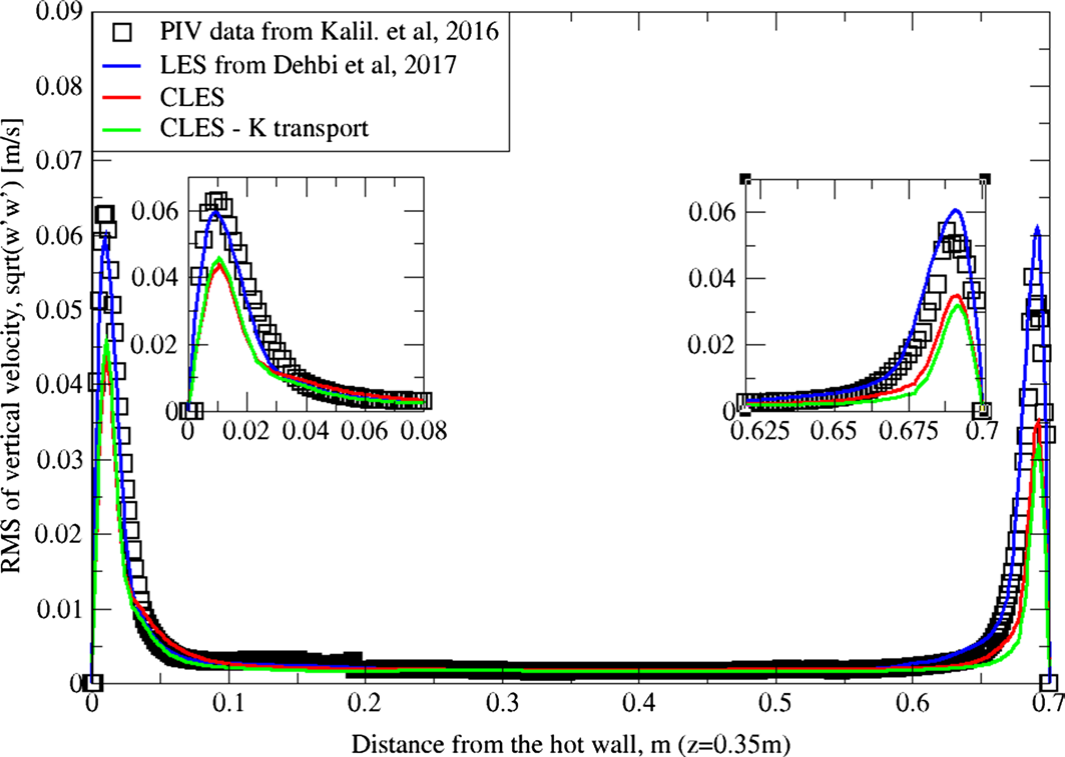
Comparison for RMS vertical velocity profiles between hot and cold walls (at z = 0.35 m) from both the standard dynamic Smagorinsky model and the dynamic model with TKE transport against reference LES and experimental databases

**Fig. 22:**
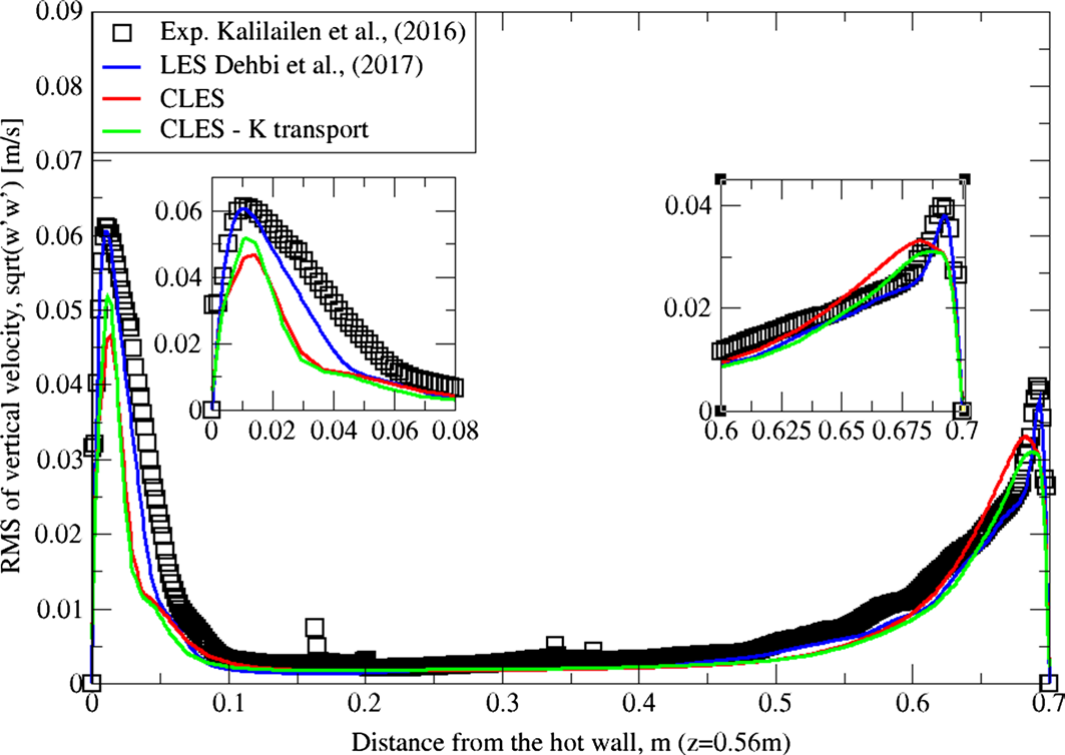
Comparison for RMS vertical velocity profiles between hot and cold walls (at z = 0.56 m) from both the standard dynamic Smagorinsky model and the dynamic model with TKE transport against reference LES and experimental databases

The reason for the good performance of the dynamic model is the accurate representation of unresolved motion scales. Unlike the standard Smagorinsky model, varying the Smagorinsky coefficient in time and space allows the turbulent viscosity to be more accurately predicted. This in turn gives a better representation for the SGS model, especially in the near-wall region where strong turbulence anisotropy is expected. It is known that the dynamic Smagorinsky model coupled with the turbulent kinetic energy (TKE) transport is the same as the standard dynamic Smagorinsky model but with a direct prediction of SGS turbulent kinetic energy provided by an additional transport equation. To show this, an additional simulation of CLES with the dynamic SGS TKE-transport model was conducted. For the sake of relevance and conciseness, we show the mean and RMS profiles of the wall-normal velocity component (i.e. ${U}_{3}$, ${u}_{3}^{^{\prime}}$ or $W, {w}^{^{\prime}}$). As in Figs. 19, 20, 21, and 22, the comparison shows that the Eulerian statistics of both models with CLES are basically the same, and therefore, the use of either model will yield the same particle depletion rates. To this end, the standard dynamic Smagorinsky model was used for the rest of the study for better efficiency.

### Lagrangian Statistics

**Fig. 23:**
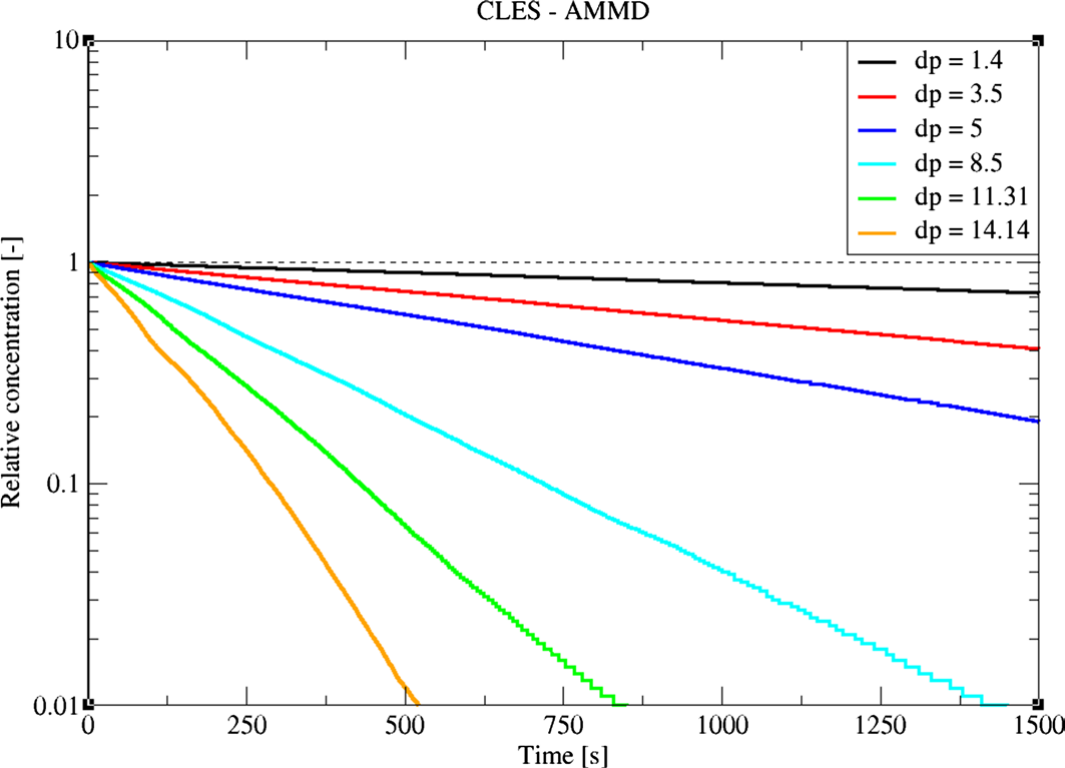
CLES predictions of relative concentration for the reported particle sizes. Each symbol represents a different particle aerodynamic mass mean diameter (AMMD)

**Fig. 24:**
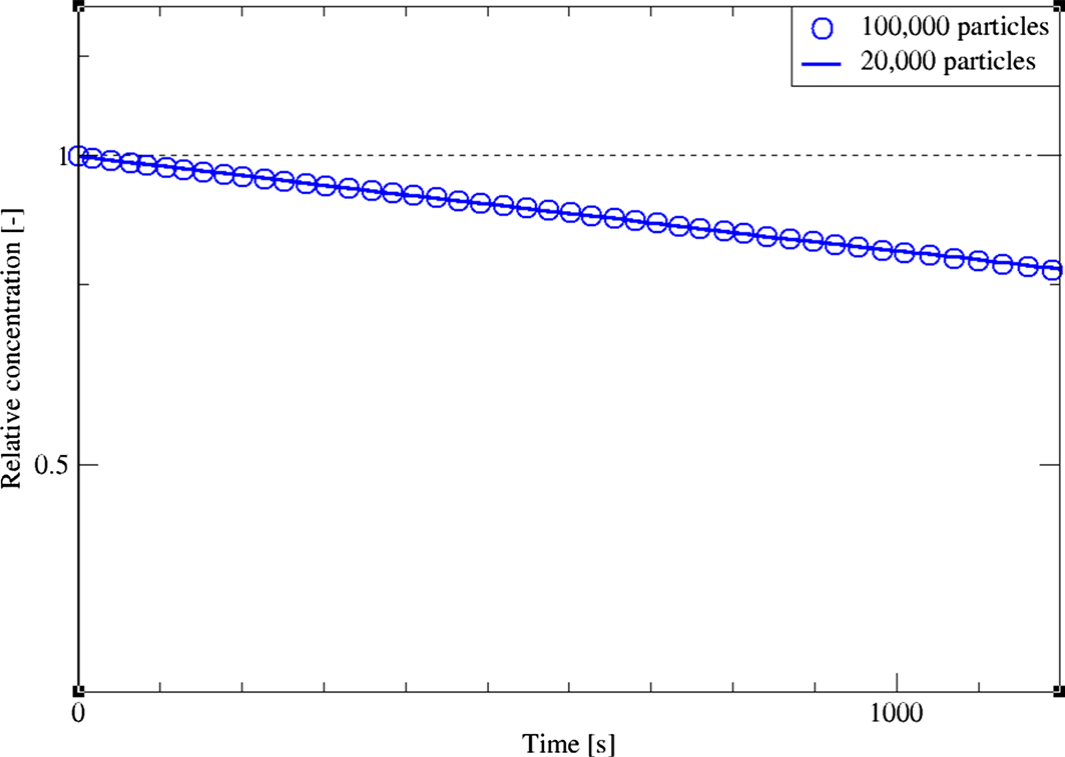
Relative concentration for two different particle counts obtained by CLES. Plots are reported for dp = 1.4 μm

In the following, we focus on the particle depletion predicted by the LES dynamic model on a coarse mesh. Following the studies by both Dehbi et al. (2017) and Kalilainen et al. (2017), we investigate the local relative concentration of airborne particles in the cavity as a function of time. As reported in Table 2, six swarms of 10^5^ particles each were considered as in Fig. 23. Since LPT is a Monte Carlo process, the solution converges to a narrow uncertainty margin when a larger sample size is used (convergence scales with N^1/2^). As a preliminary simulation, the two sample sizes of 10^5^ and 2 × 10^4^ particles didn’t show a significant change in results (Fig. 24). However, it must be stressed that in such flow configuration, small deviations get amplified over time, and therefore, deposition time can be huge for small particles. For this reason, the sample comprising 10^5^ particles was considered sufficiently large for obtaining credible results.

Since particle depletion rate takes place at a small rate, the following results are obtained in a statistically developing manner. As shown in Fig. 19, local particle concentrations in time are reported where only the airborne particles are accounted for at each time, normalized by particle concentration at t = 0. Following the analysis of both Kalilainen et al. (2016) and Dehbi et al. (2017), the stirred settling model (Hinds 1999) is used as another reference to estimate particle depletion rate inside the cavity. The model assumes a perfect spatial uniformity of particle distribution at any moment, which is approximately the case if a mechanical tool was used to “stir” the particles. The model also assumes that particles have a net velocity equal to the gravitational terminal speed ${V}_{TS}$. With such definition, temporally local concentration has an exponential relation with time as follows:

19 $$C\left( t \right) = C\left( 0 \right) {\text{exp}}\left( {\frac{ - t}{{{\raise0.7ex\hbox{$L$} \!\mathord{\left/ {\vphantom {L {V_{TS} }}}\right.\kern-\nulldelimiterspace} \!\lower0.7ex\hbox{${V_{TS} }$}}}}} \right) = C\left( 0 \right) {\text{exp}}\left( {\frac{ - t}{{{\raise0.7ex\hbox{$L$} \!\mathord{\left/ {\vphantom {L {\tau_{p} g}}}\right.\kern-\nulldelimiterspace} \!\lower0.7ex\hbox{${\tau_{p} g}$}}}}} \right)$$

**Fig. 25:**
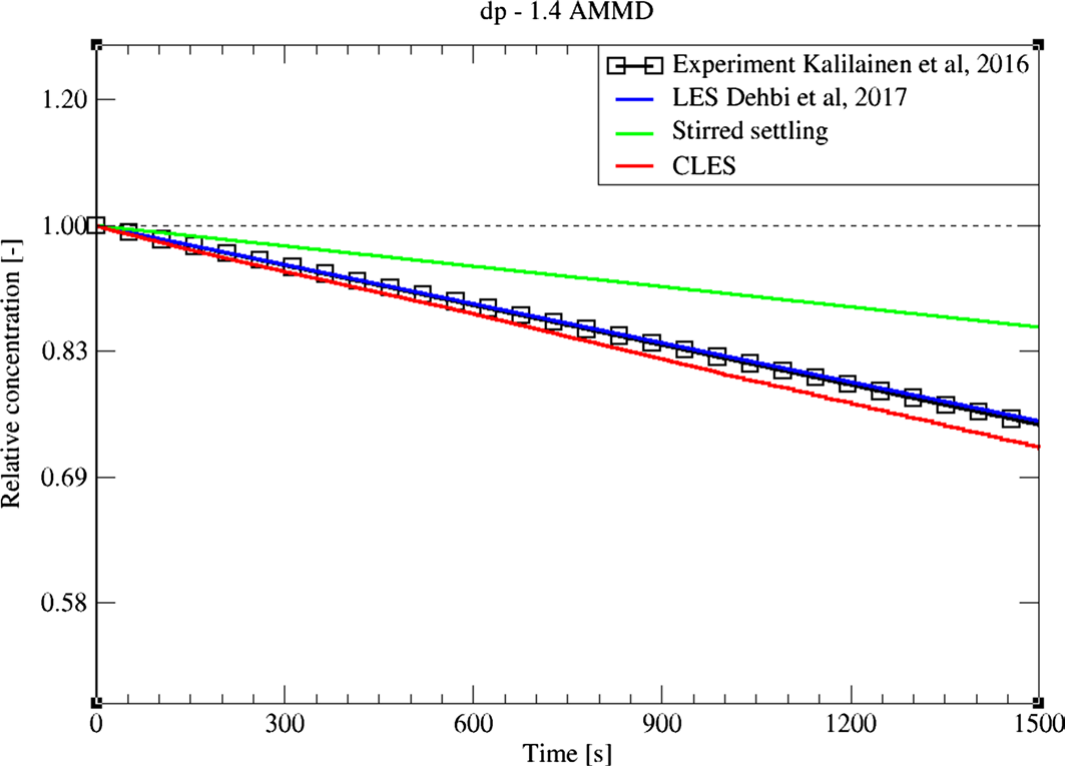
Comparison for particle relative concentration of dp = 1.4 μm versus time. Predictions by CLES are plotted against all of LES from Dehbi et al. (2017), experimental data by Kalilainen et al. (2016), and the stirred settling model (Hinds 1999)

Despite providing a good picture about inertial particle depletion, the stirred settling model is considered a rough approximation for relatively small inertia particles where the timescale of the particle is comparable to the typical eddy fluctuation in the domain (Fig. 25). This is due to the rapid response of low-inertia particles to the carrier flow, and therefore, the model assumption of terminal speed does not hold. This finding was confirmed in the study of Dehbi et al. (2017) using well-resolved LES.

**Fig. 26:**
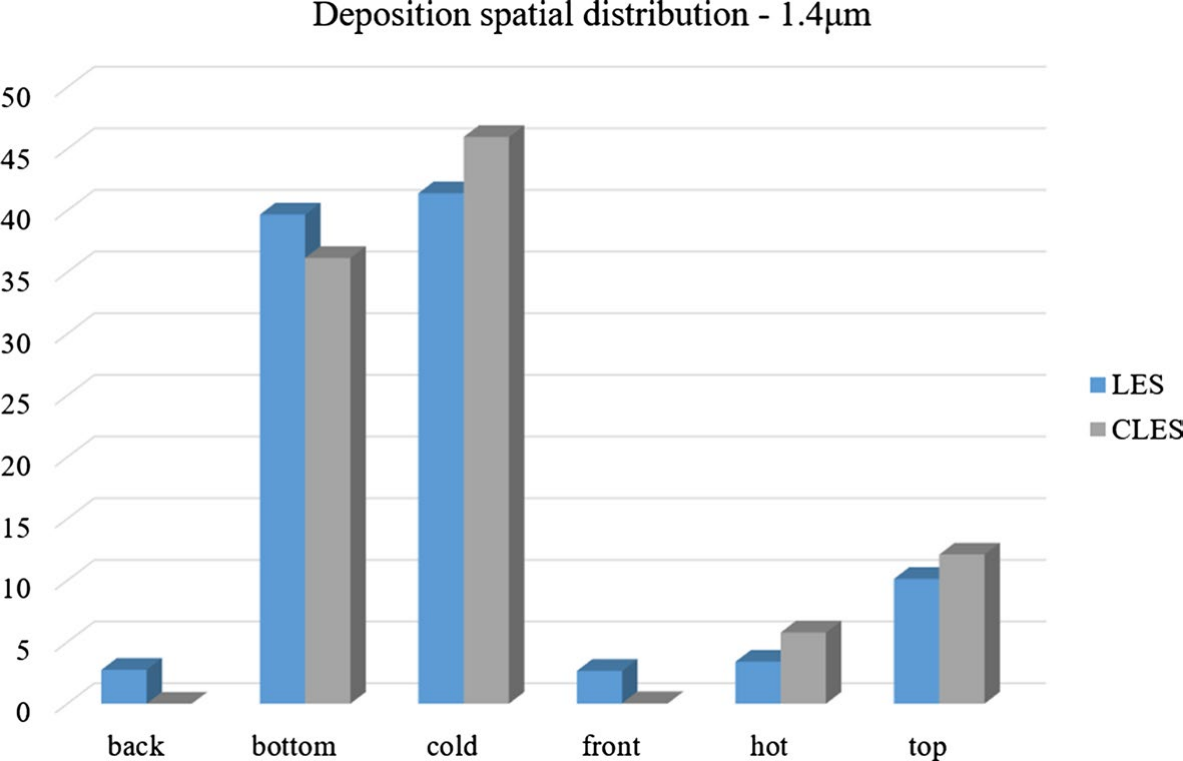
Representation of spatially deposited particles on each wall at t = 700 s. Predictions by CLES for particle size dp = 1.4 μm are compared against LES from Dehbi et al. (2017)

Comparing the predictions of CLES against both of LES and the reference experimental data, it can be seen that there is a small deviation in relative concentration for 1.4 μm particles (Fig. 25). However, this deviation is not significant given that depletion rates are plotted over a large time interval. Looking at the spatial distribution of the deposited particles of size 1.4 μm (Fig. 26), it could be shown that SGS has also little effect on the deposition pattern over the cavity walls. As can be seen from the histogram representation, the spatial distribution of deposited particles is in line with the reference LES results with less than 5% deviation.

**Fig. 27:**
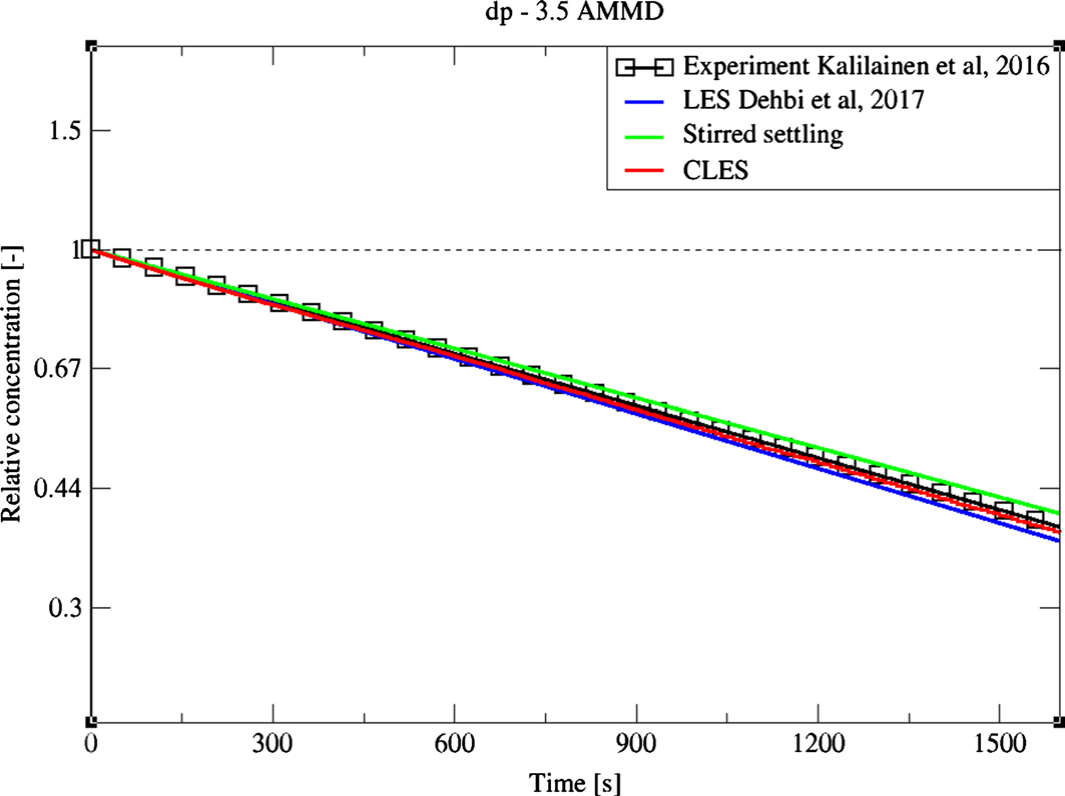
Comparison for particle relative concentration of dp = 3.5 μm versus time. Predictions by CLES are plotted against all of LES from Dehbi et al. (2017), experimental data by Kalilainen et al. (2016), and the stirred settling model (Hinds 1999)

**Fig. 28:**
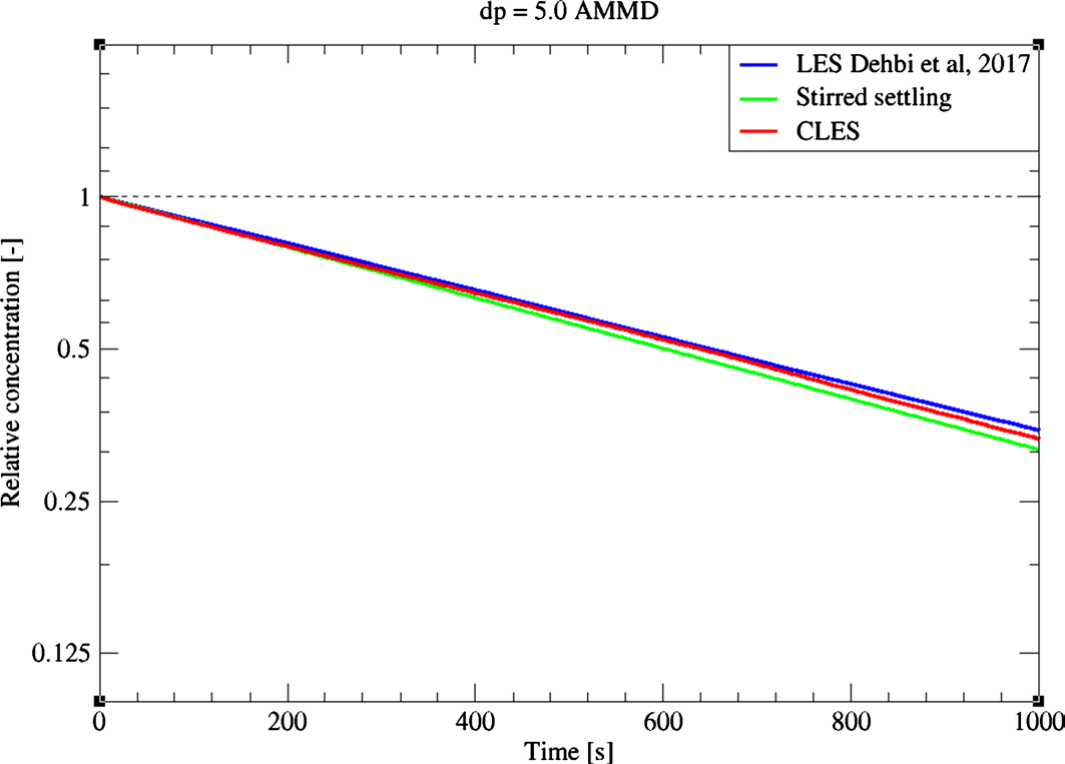
Comparison for particle relative concentration of dp = 5.0 μm versus time. Predictions by CLES are plotted against both of LES from Dehbi et al. (2017) and the stirred settling model (Hinds 1999)

**Fig. 29:**
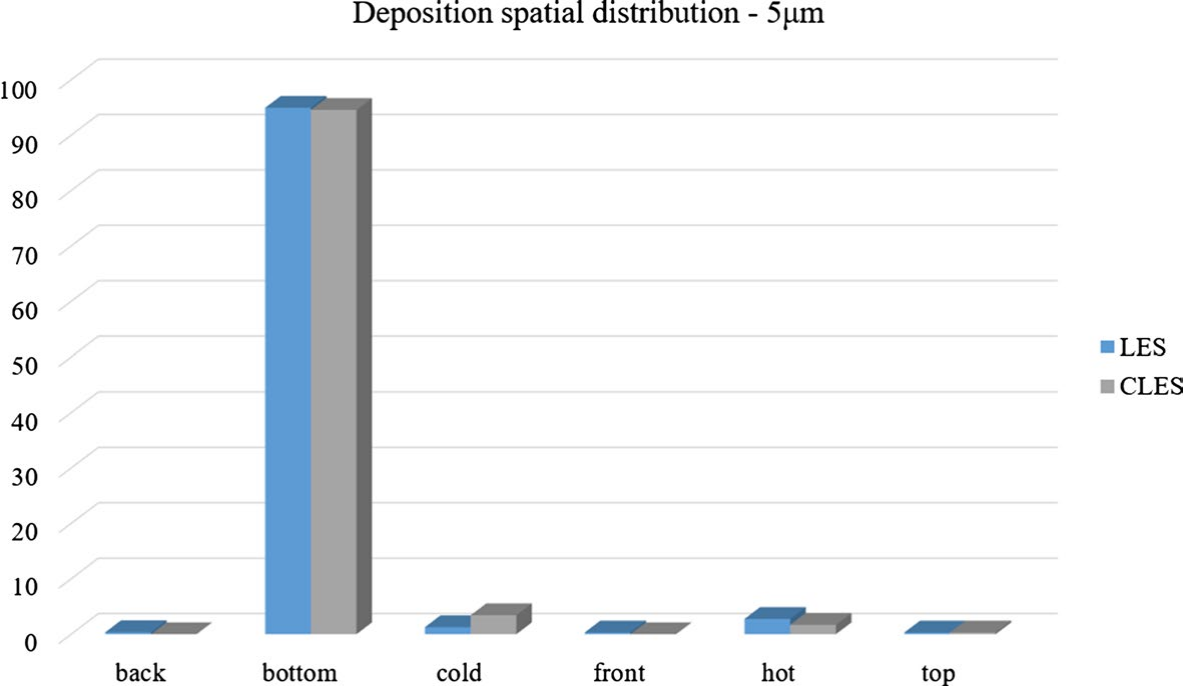
Representation of spatially deposited particles on each wall at t = 700 s. Predictions by CLES for particle size dp = 5 μm are compared against LES from Dehbi et al. (2017)

For a bigger particle diameter i.e. dp = 3.5, 5 μm, it could be noticed from Figs. 27 and 28 that CLES prediction is still in very good agreement with both LES an experimental data. As mentioned above, it could also be seen that the stirred settling model approaches the experimental trend as particle size gets bigger (Fig. 27). Similar to the spatial deposition for 1.4 μm particles, it can be seen from Fig. 29 that for higher-inertia particles i.e. 5 μm, gravity force is more dominant than both drag and thermophoretic forces. This can be inferred from the better agreement in deposition pattern between CLES and the reference LES relative to the smaller-inertia particle size.

**Fig. 30:**
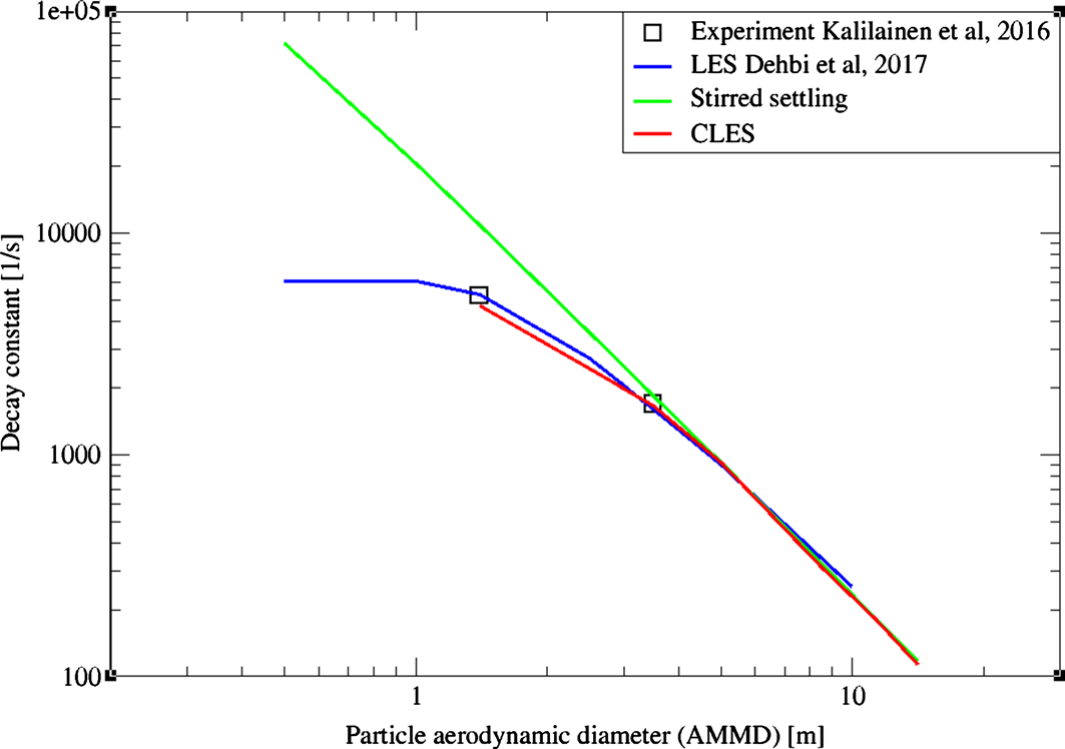
Comparison for particle decay constant obtained by CLES against all of experimental and LES data as well as the stirred settling model

In order to have a more quantitative picture, the decay constant was calculated for each particle diameter against LES predictions. The decay constant ${\beta }_{D}$ is defined as the inverse of time constant for particle removal (where time constant is computed from ${\uplambda } = {{ - ln\left( {\frac{N\left( t \right)}{{N\left( 0 \right)}}} \right)} \mathord{\left/ {\vphantom {{ - ln\left( {\frac{N\left( t \right)}{{N\left( 0 \right)}}} \right)} {time}}} \right. \kern-\nulldelimiterspace} {time}}$). As can be seen from Fig. 30, such comparison reveals that due to SGS effects the decay constant is slightly underestimated below 3.5 μm. In contrast, bigger particles decay rates align perfectly with the correct depletion rate since they are less affected by turbulent fluctuations.

## Conclusion

The flow field inside a three-dimensional, wall-bounded, differentially heated cavity (DHC) has been investigated using Large Eddy Simulations on a mesh significantly coarser than used in the previous research at Rayleigh number of Ra = 10^9^. In particular, the LES dynamic Smagorinsky model have been used. Wall-to-wall radiation effects have been implicitly taken into account by imposing the measured temperature profiles of bottom and top walls as Dirichlet boundary conditions. A quantitative analysis is reported for the fluid flow where first and second-moment statistics are reported. The results are compared against both previous well-resolved LES by Dehbi et al. (2017) and the experimental database by Kalilainen et al. (2016).

It was shown that the mean flow results of coarse LES are globally in very good agreement with reference LES and experimental measurements at a fraction of CPU cost relative to LES by Dehbi et al. (2017). However, second moment Eulerian statistics are underestimated due to the low mesh resolution in the boundary layer region. Despite the deviations in higher moment statistics, the obtained results agree on both qualitative and quantitative grounds with the results obtained by LES of Dehbi et al. (2017) and the experimental measurements of Kalilainen et al. (2016).

In a second step, the fluid flow was used for particle tracking in an Euler/Lagrange frame. In a systematic study, six swarms of 10^5^ particles each were computed to investigate a wide range of particle aerodynamic diameters dp = 1.4–14 μm. In particular, predictions of particle depletion rates for dp = 1.4, 3.5 μm obtained by coarse LES were compared to reference LES and experimental databases. Results show that SGS motions have little effect on particle depletion rates—especially for inertial particles (i.e. dp > 1.4 μm).

Good performance of coarse dynamic LES simulation with less than 0.125 million cells compared to the 2.4 million-cell-mesh of well-resolved LES confirms that for the cavity simulation LES with the right SGS model can still be used to predict accurately the motion of a specific band of particle sizes. Authors consider the finding of the present study a promising step for further complex particulate flow investigation using coarse LES.

## Abbreviations

DNS: Direct numerical simulation
LES: Large eddy simulation
CLES: Coarse large eddy simulation
WRLES: Wall-resolved LES
RANS: Reynolds-averaged Navier stokes
IRC: Intermediate realistic conditions
AMMD: Aerodynamic mass median diameter
LPT: Lagrangian particle tracking

## List of Symbols

L: Cavity length [m]
${k}_{f}$: Fluid thermal conductivity [W/(m⋅K)]
${k}_{p}$: Particle thermal conductivity [W/(m⋅K)]
$\lambda$: Mean free path [m]
$$\alpha = \frac{{k}_{f}}{{\rho c}_{p}}$$: Thermal diffusivity [m^2^/s]
$\upsilon = \frac{\mu }{\rho }$: Momentum kinematic diffusivity [m^2^/s]
${\tau }_{w}$: Wall shear stress [${m}^{2}/{s}^{2}$]
${\rho }_{f}$: Fluid density [kg/${m}^{3}$]
${\rho }_{p}$: Particle density [kg/${m}^{3}$]
$${d}_{p}$$: Particle diameter [$$m$$]
${d}_{\rm AMMD}= \sqrt{\frac{{\rho }_{p}}{{\rho }_{{H}_{2}O}}{{d}_{p}}^{2}}$: Particle aerodynamic diameter [$m$]
$${\upsilon }_{t}$$: Turbulent diffusivity [m^2^/s]
$$\rm Pr$$ = $$\frac{\upsilon }{\alpha }$$: Prandtl number
${\rm Pr}_{t}$ = $\frac{{\upsilon }_{t}}{{\alpha }_{t}}$: Turbulent Prandtl number
$\beta$: Thermal expansion coefficient [1/K]
$${\rm Ra}= \frac{g \beta \Delta T {L}^{3}}{\upsilon \alpha }$$: Rayleigh number
${U}_{i}$: Dimensional fluid velocity vector [m/s]
${V}_{p,i}$: Dimensional particle velocity vector [m/s]
$${g}_{i}$$: Gravitational vector [$$m/{s}^{2}$$]
T: Temperature [K]
$k$: Turbulent kinetic energy [${m}^{2}/{s}^{2}$]
$\underset{\_}{{u}_{i}{u}_{j}}$: Reynolds stress tensor [${m}^{2}/{s}^{2}$]
$\underset{\_}{{u}_{i}\theta }$: Turbulent heat flux [mK/s]
$${T}_{\rm ref}$$: Reference temperature [K]
${\rho }_{\rm ref}$: Reference density [kg/${m}^{3}$]
${\tau }_{f}= \frac{L}{{V}_{r}}$: Circulation timescale [s], or fluid time-scale
$${\tau }_{p}$$: Particle timescale [s]
$${V}_{r }= \frac{\alpha \sqrt{Ra}}{L}$$: Circulation speed [m/s]
${V}_{TS }= {\tau }_{p} {g}_{i}$: Particle gravitational terminal speed [m/s]
$$St = \frac{{\tau }_{p}}{{\tau }_{f}}$$: Stokes number
$${u}_{\tau }= \sqrt{\frac{{\tau }_{w}}{\rho }}$$: Friction velocity [m/s]
${\rm Re}=\frac{Vr L}{\upsilon }$: Reynolds number based on circulation velocity
${C}_{c}$: Cunningham slip-correction factor [-]
$C(t)$: Local particle concentration in time [-]
$C(0)$: Initial particle concentration [-]
$${c}_{p}$$: Heat capacity [J/K]
${y}^{+}= \frac{y {u}_{\tau }}{\upsilon }$: Non-dimensional wall distance
$$\Delta T$$: Temperature difference
$\Delta {X}_{i}$: Cell base size
